# Compartment-Specific and Sequential Role of MyD88 and CARD9 in Chemokine Induction and Innate Defense during Respiratory Fungal Infection

**DOI:** 10.1371/journal.ppat.1004589

**Published:** 2015-01-26

**Authors:** Anupam Jhingran, Shinji Kasahara, Kelly M. Shepardson, Beth A. Fallert Junecko, Lena J. Heung, Debra K. Kumasaka, Sue E. Knoblaugh, Xin Lin, Barbara I. Kazmierczak, Todd A. Reinhart, Robert A. Cramer, Tobias M. Hohl

**Affiliations:** 1 Infectious Disease Service, Department of Medicine, Memorial Sloan Kettering Cancer Center, New York, New York, United States of America; 2 Department of Microbiology and Immunology, Geisel School of Medicine at Dartmouth University, Hanover, New Hampshire, United States of America; 3 Department of Infectious Diseases and Microbiology, University of Pittsburgh, Pittsburgh, Pennsylvania, United States of America; 4 Division of Human Biology, Fred Hutchinson Cancer Research Center, Seattle, Washington, United States of America; 5 Comparative Medicine Shared Resources, Fred Hutchinson Cancer Research Center, Seattle, Washington, United States of America; 6 Department of Molecular and Cellular Oncology, University of Texas, MD Anderson Cancer Center, Houston, Texas, United States of America; 7 Department of Medicine and Department of Microbial Pathogenesis, Yale University School of Medicine, New Haven, Connecticut, United States of America; 8 Immunology Program, Sloan-Kettering Institute, Memorial Sloan Kettering Cancer Center, New York, New York, United States of America; University of Wisconsin-Madison, UNITED STATES

## Abstract

*Aspergillus fumigatus* forms ubiquitous airborne conidia that humans inhale on a daily basis. Although respiratory fungal infection activates the adaptor proteins CARD9 and MyD88 via C-type lectin, Toll-like, and interleukin-1 family receptor signals, defining the temporal and spatial pattern of MyD88- and CARD9-coupled signals in immune activation and fungal clearance has been difficult to achieve. Herein, we demonstrate that MyD88 and CARD9 act in two discrete phases and in two cellular compartments to direct chemokine- and neutrophil-dependent host defense. The first phase depends on MyD88 signaling because genetic deletion of MyD88 leads to delayed induction of the neutrophil chemokines CXCL1 and CXCL5, delayed neutrophil lung trafficking, and fatal pulmonary damage at the onset of respiratory fungal infection. MyD88 expression in lung epithelial cells restores rapid chemokine induction and neutrophil recruitment via interleukin-1 receptor signaling. Exogenous CXCL1 administration reverses murine mortality in MyD88-deficient mice. The second phase depends predominately on CARD9 signaling because genetic deletion of CARD9 in radiosensitive hematopoietic cells interrupts CXCL1 and CXCL2 production and lung neutrophil recruitment beyond the initial MyD88-dependent phase. Using a CXCL2 reporter mouse, we show that lung-infiltrating neutrophils represent the major cellular source of CXCL2 during CARD9-dependent recruitment. Although neutrophil-intrinsic MyD88 and CARD9 function are dispensable for neutrophil conidial uptake and killing in the lung, global deletion of both adaptor proteins triggers rapidly progressive invasive disease when mice are challenged with an inoculum that is sub-lethal for single adapter protein knockout mice. Our findings demonstrate that distinct signal transduction pathways in the respiratory epithelium and hematopoietic compartment partially overlap to ensure optimal chemokine induction, neutrophil recruitment, and fungal clearance within the respiratory tract.

## Author Summary

Our understanding of how epithelial and hematopoietic cells in the lung coordinate immunity against inhaled fungal conidia (spores) remains limited. The mold *Aspergillus fumigatus* is a major cause of infectious mortality in immune compromised patients. Host defense against *A. fumigatus* involves the activation of two host signal transducers, MyD88 and CARD9, leading to neutrophil recruitment to the infection site. In this study, we define how MyD88- and CARD9-coupled signals operate in epithelial and hematopoietic compartments to regulate neutrophil-mediated defense against *A. fumigatus*. Our studies support a two-stage model in which MyD88 activation in epithelial cells, via the interleukin-1 receptor, supports the rapid induction of neutrophil-recruiting chemokines. This process is essential for the first phase of neutrophil recruitment. Mortality observed in MyD88-deficient mice can be significantly reversed by administration of a chemokine termed CXCL1 to infected airways. The second phase of neutrophil recruitment is initiated by CARD9 signaling in hematopoietic cells. Loss of both phases of chemokine induction and neutrophil recruitment dramatically increases murine susceptibility to tissue-invasive disease. In sum, our study defines a temporal sequence of events, initiated by interleukin-1 receptor/MyD88 signaling in the pulmonary epithelium and propagated by CARD9 signaling in hematopoietic cells, that induces protective immunity against inhaled fungal conidia.

## Introduction

*Aspergillus fumigatus* is a leading cause of infectious morbidity and mortality in patients with hematologic malignancies, allogeneic stem cell transplant recipients and in patients receiving immune suppressive therapies [1,2]. Airborne conidia (asexual spores) represent the infectious propagules that humans inhale daily. Due to their small size (∼2–3 μm diameter), conidia can bypass mucociliary clearance and reach terminal airways [3,4]. In immune competent individuals, lifelong asymptomatic clearance of inhaled conidia prevents the formation of tissue-invasive hyphae.

Neutrophils represent essential effector phagocytes that are rapidly recruited to infected airways and act against *A.fumigatus* conidia and hyphae [5–7]. Neutrophil recruitment to the portal of infection depends on rapid chemokine induction, with a prominent role for ELR^+^ CXC chemokines and their receptors in this process. In mice, antibody-mediated blockade [8] or genetic deficiency of the chemokine receptor CXCR2 [7] results in delayed neutrophil airway recruitment and the development of invasive aspergillosis (IA). Consistent with this notion, overexpression of a CXCR2 ligand (i.e. CXCL1/KC) in the murine lung improves outcomes in neutropenic mice infected with *A. fumigatus* [9].

Although a number of signaling pathways have been linked to the induction of CXCR2 ligands (i.e. the CXCL chemokines CXCL1/KC, CXCL2/MIP-2, and CXCL5/LIX) and to protective innate immune responses, the events that couple fungal recognition to neutrophil recruitment remain incompletely defined. Conidial swelling, the first step in fungal germination to filamentous hyphae, leads to stage-specific exposure of ligands that activate C-type lectin receptor (CLR) [10–12], NOD-like receptor (NLR) [13], Toll-like-receptor (TLR), and interleukin-1-receptor (IL-1R) signaling *in vitro* and *in vivo* [14,15].

The CLRs Dectin-1 (i.e. CLEC7A) [16,17] and Dectin-2/-3 (i.e. CLEC4N/CLEC4D) [18,19] recognize fungal β-glucan and α-mannan respectively, and transduce signals via spleen tyrosine kinase (Syk), protein kinase C-δ, and caspase recruitment domain-containing protein 9 (CARD9) [20–22]. The latter forms a trimeric complex with Bcl10 (i.e. B cell lymphoma/leukemia 10) and MALT1 (mucosa associated lymphoid tissue lymphoma translocation protein 1) and activates NF-κB-dependent transcription, for example of the *tnf* and *il1β* genes [20,23]. Both Dectin-1 and CARD9 have been linked to the induction of CXCR2 ligands during respiratory *A. fumigatus* infection [14,23]. *In vitro, A. fumigatus* can induce Syk-dependent assembly of cytoplasmic multiprotein complexes termed inflammasomes that incorporate NLRP3 (Nod-like receptor family, pyrin domain-containing 3) and the scaffold protein ASC (Apoptosis-associated speck-like protein containing a CARD) and result in caspase-dependent proteolytic cleavage of pro-IL-1β into active IL-1β [13,24,25]. In vivo, respiratory *A. fumigatus* and systemic *Candida albicans* infection induce IL-1α and IL-1β production [14,23,26], linking fungal recognition to IL-1 receptor type I (IL-1R) signaling.

IL-1R and most TLR superfamily members activate the signal transducer MyD88 (Myeloid differentiation primary response gene 88). Similar to CARD9, MyD88 activation following *A. fumigatus* challenge has been linked to the induction of NF-κB-dependent production of CXCR2 ligands *in vitro* and *in vivo* [10,23,27–29]. Although TLR2- and TLR4-deficient macrophages and dendritic cells show defects in the production of CXCR2 ligands *in vitro* [10,11,30], the relative contribution of TLR-MyD88 versus IL1R-MyD88 signaling in neutrophil recruitment and in protective innate immunity in the lung following respiratory *A. fumigatus* challenge remains to be elucidated.

During respiratory fungal challenge, the mechanism by which the host integrates signals from CLR/CARD9, IL-1R1/MyD88, and TLR/MyD88 pathways to achieve rapid chemokine-dependent influx of effector phagocytes and conidial clearance remains undefined. One possible scenario is that CARD9- and MyD88-dependent signals act within the same cellular compartment and in the same time frame for optimal chemokine induction, as has been demonstrated for Dectin-1/CARD9 and TLR2/MyD88-dependent macrophage TNF and IL-12 release in response to the model fungal particle zymosan [21,31]. In a second scenario, MyD88- and CARD9-coupled signals may act in distinct cellular compartments in the lung within the same time phase post-infection (p.i.) to induce chemokines and coordinate innate immune responses. The latter scenario is exemplified by *Pseudomonas aeruginosa*- or *Legionella pneumophila*-induced IL-1α/IL-1β production by hematopoietic cells; this process, in turn, rapidly activates IL-1R1 signaling in pulmonary epithelial cells to promote airway neutrophil influx [32–34]. However, since gram-negative bacteria associated with pneumonia are not known to induce CLR/CARD9 signaling *in vivo*, conclusions derived from the above studies may not extrapolate to respiratory fungal infection models. A common prediction of the first and second scenario is that a defect in either signaling pathway would diminish chemokine induction and neutrophil recruitment in the same time period p.i. These scenarios could be distinguished by identifying common or distinct cellular compartments in which the signaling pathways operate.

Alternatively, CLR/CARD9 and TLR/MyD88 or IL1R1/MyD88 signaling pathways may act primarily in temporally distinct phases, either in the same or in distinct cellular compartments, creating two additional scenarios. In these scenarios, each protein would mediate a distinct phase of chemokine induction and neutrophil recruitment that is largely independent of the phase induced by the other signaling adapter. For example, MyD88- and type I interferon signaling induce sequential phases of CCL2/MCP-1, a chemokine that regulates inflammatory monocyte trafficking during systemic listeriosis [35].

In this study, we demonstrate that IL1R1/MyD88 signaling plays a crucial role in innate host defense by mediating rapid CXCL chemokine induction and airway neutrophil recruitment during respiratory fungal infection. This initial phase of chemokine induction and effector cell recruitment occurs independently of CARD9 signaling and depends on IL-1R and MyD88 expression within lung epithelial cells. The mortality defect observed in MyD88^(−/−)^ mice can be significantly ameliorated by administration of a single dose of recombinant CXCL1 at the onset of infection, consistent with the notion that MyD88-coupled signals act to orchestrate neutrophil recruitment. In contrast, CARD9 signaling within hematopoietic cells mediates CXCL chemokine induction and neutrophil recruitment in the second phase, with neutrophils representing a major cellular source of CXCL2. Since cell-intrinsic MyD88 and CARD9 expression in neutrophils are dispensable for the induction of neutrophil conidiacidal activity, our data indicate that MyD88- and CARD9-coupled signals act primarily to orchestrate sequential phases of neutrophil recruitment during respiratory *A. fumigatus* infection. Thus, our data illustrate an essential role for lung epithelial cells in innate antifungal immunity and delineate compartment-specific and additive roles for MyD88 and CARD9 signaling that collectively regulate chemokine induction and neutrophil trafficking during pulmonary infection with *A. fumigatus*.

## Results

### MyD88 mediates murine survival after respiratory *A. fumigatus* challenge

**Figure 1:**
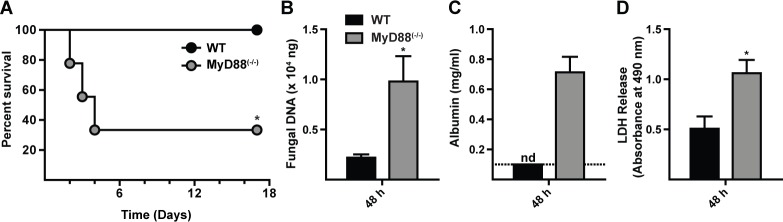
MyD88 is critical for survival, fungal clearance, and lung integrity during *A. fumigatus* challenge. WT and MyD88^(−/−)^ mice were challenged with 7 × 10^7^ conidia and (A) monitored for survival (Kaplan-Meier survival plot of WT (black circles; n = 9) and MyD88^(−/−)^ (grey circles; n = 9) mice), assayed for (B) lung fungal burden, (C) BALF albumin, and (D) BALF LDH levels at 48 h p.i. (A) One of three experiments shown. (B-D) The bar graphs show mean (+SEM) values from an experiment with 6–7 mice per genotype. (C) nd = none detected. The value was below the limit of detection of the albumin assay (dashed line).

To define the role of MyD88 during respiratory fungal challenge, MyD88^(−/−)^ and C57BL/6 control mice were challenged with 7 × 10^7^ *A. fumigatus* Af293 conidia and monitored for survival. The median survival time for MyD88^(−/−)^ mice was 3 days, while all C57BL/6 mice survived the 17 day observation period (Fig. 1A). Mortality in MyD88^(−/−)^ mice correlated with an increased fungal burden (Fig. 1B) and greater lung damage, as measured by bronchoalveolar lavage fluid (BALF) albumin (Fig. 1C) and lactate dehydrogenase (LDH) release (Fig. 1D), compared to control mice at 48 h p.i.

Histopathologic analysis demonstrated differences in the pattern of inflammation between the two groups at 48 h p.i. (S1A-S1B Fig. in S1 Text). Within WT lung sections moderate to severe, multifocal to coalescing inflammation was widespread (S1A and S1C Fig. in S1 Text). Although inflammatory foci in MyD88^(−/−)^ lung sections were not as widespread as in control mice, the foci were admixed with cellular debris indicative of severe tissue necrosis (S1B and S1D Fig. in S1 Text), consistent with the development of necrotizing fungal bronchopneumonia. Although MyD88^(−/−)^ mice exhibited a higher fungal burden in the lung than WT mice, overt hyphal tissue invasion was not apparent in either group. In sum, these results indicate that early MyD88 function is essential for effective fungal clearance and prevents tissue damage that compromises murine survival.

### MyD88 is required for the first phase of neutrophil recruitment but not for neutrophil effector functions against conidia

**Figure 2:**
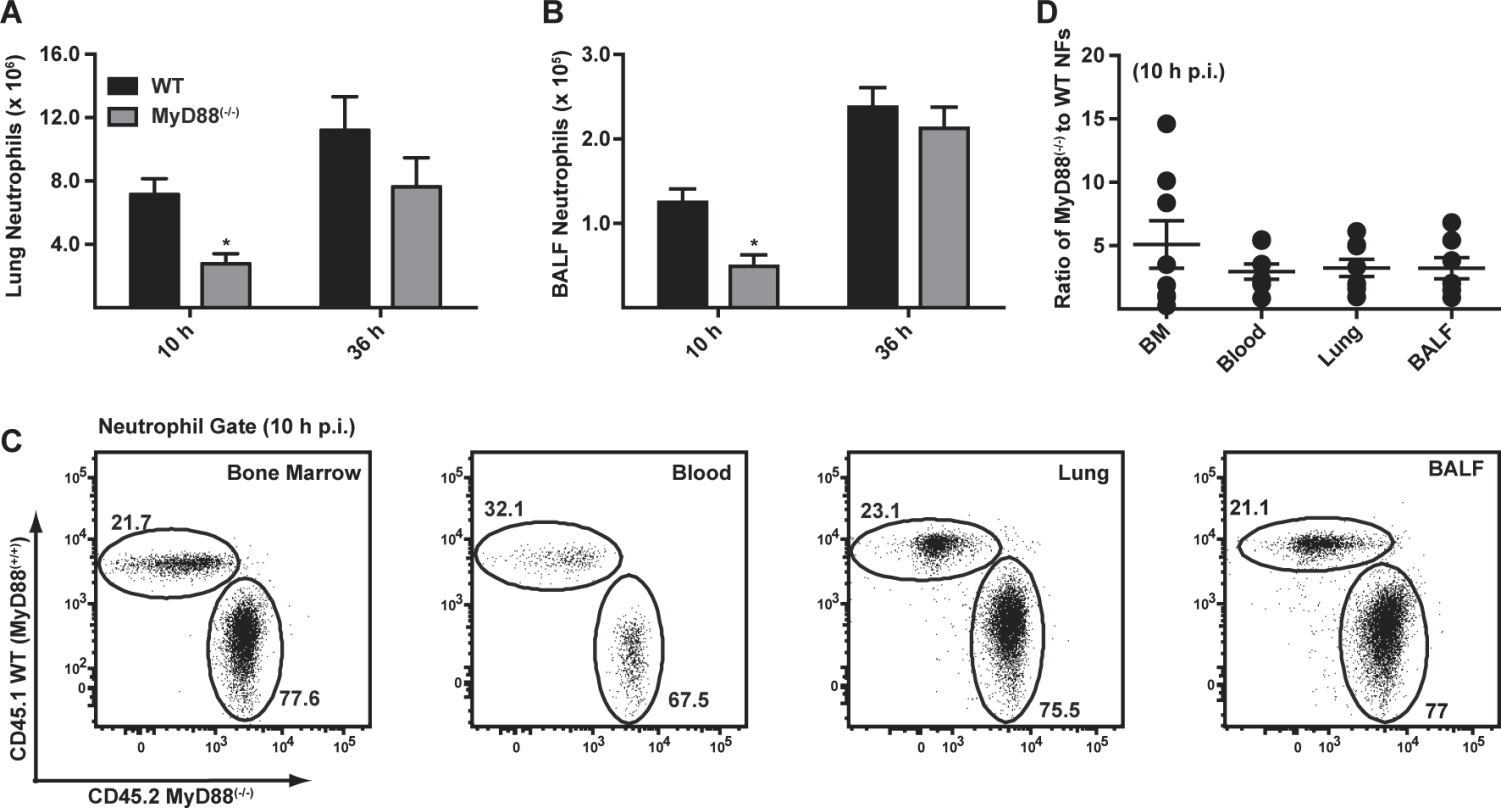
MyD88 mediates the first stage of neutrophil recruitment to the lung. (A-B) The bar graphs show the mean (+SEM) (A) lung and (B) BALF neutrophil numbers 10 h and 36 h p.i. in C57BL/6 (black bars) and MyD88^(−/−)^ (grey bars) mice challenged with 3 × 10^7^ conidia. Data were pooled from 3 independent experiments and include 11–14 mice per group per time point. (C) The flowplots show the frequencies of CD45.1^+^ MyD88^(+/+)^ and CD45.2^+^ MyD88^(−/−)^ neutrophils in the BM, blood, lung and BALF in a representative bone marrow chimeric mouse 10 h p.i. with 3 × 10^7^ conidia. CD45.1^+^CD45.2^+^ recipient mice were lethally irradiated and reconstituted with a 3:1 ratio of CD45.2^+^ MyD88^(−/−)^ to CD45.1^+^ MyD88^(+/+)^ BM cells. The plots were gated on CD11b^+^Ly6G^+^ neutrophils. (D) The graph shows the mean ratio (±SEM) of MyD88^(−/−)^ to WT neutrophils (NFs) in BM, blood, lung and BALF pooled from two experiments with 7 mice.

To dissect the mechanism by which MyD88 mediates early host protection against *A. fumigatus*, we compared the recruitment and functional properties of neutrophils in MyD88^(−/−)^ and C57BL/6 mice. Neutrophil numbers in the lung and BALF were consistently decreased in MyD88^(−/−)^ mice compared to C57BL/6 controls at 10 h p.i. (Fig. 2A & 2B) with similar neutrophil numbers observed in both compartments at 36 h p.i., consistent with an initial MyD88-dependent phase of cell recruitment following respiratory *A. fumigatus* infection. In contrast, the number of lung monocytes, lung and alveolar macrophages, and lung CD11b^+^ DCs was similar in MyD88^(−/−)^ and control mice at 10 h p.i. (S2A-S2D Fig. in S1 Text; see S2E-S2G Fig. in S1 Text for leukocyte gating strategy).

Fluorescent *A. fumigatus* reporter (FLARE) conidia (S2 Text) [23] contain two fluorophores (dsRed and Alexa Fluor 633) that enable lung leukocytes to be distinguished on the basis of conidial uptake and killing because dsRed fluorescence, but not Alexa Fluor 633 fluorescence, is extinguished when conidia are killed. Using FLARE conidia, we observed that neutrophil fungal cell uptake in MyD88^(−/−)^ mice was defective at 10 h p.i. (S3A-S3E Fig. in S1 Text). In MyD88^(−/−)^ mice, a lower frequency of lung neutrophils internalized fungal cells at 10 h p.i. compared to lung neutrophils in WT mice (S3B and S3D Fig. in S1 Text). Furthermore, the frequency of live fungal cells within neutrophils, corrected for fungal cell uptake, was higher in neutrophils in MyD88^(−/−)^ mice compared to neutrophils in control mice at 10 h p.i. (S3C and S3E Fig. in S1 Text). Differences in neutrophil fungal cell uptake and killing in MyD88^(−/−)^ and control mice were no longer apparent at 36 h p.i. (S3B-S3E Fig. in S1 Text), suggesting that MyD88-dependent differences in neutrophil recruitment underlie the apparent reduction in phagocytic and conidiacidal activity observed at 10 h p.i.

To determine whether cell-intrinsic MyD88 function in neutrophils impacts their survival, trafficking, and effector functions, we generated mixed bone marrow (BM) chimeric mice (CD45.2^+^ MyD88^(−/−)^ and CD45.1^+^ WT MyD88^(+/+)^ BM cells injected into lethally irradiated CD45.1^+^CD45.2^+^ MyD88^(+/+)^ recipients) and compared the behavior of MyD88^(−/−)^ and MyD88^(+/+)^ neutrophils during respiratory fungal infection. The relative frequency of CD45.1^+^ MyD88^(+/+)^ and CD45.2^+^ MyD88^(−/−)^ neutrophils was unchanged as neutrophils exited the BM, entered the circulation, and trafficked to the lung and BALF 10 h p.i. (Fig. 2C-2D). These data indicate that neutrophil-intrinsic MyD88 is dispensable for neutrophil trafficking and survival during respiratory fungal infection.

Using the same experimental set-up, we next compared conidial uptake and killing by MyD88^(−/−)^ and MyD88^(+/+)^ neutrophils within the same lung, eliminating differences in cell trafficking and in inflammation observed in MyD88^(−/−)^ and in C57BL/6 mice. Conidial uptake by and conidial viability in lung neutrophils was statistically indistinguishable in MyD88^(−/−)^ and MyD88^(+/+)^ counterparts isolated from BM chimeric mice (S4A-S4B Fig. in S1 Text). Furthermore, *in vitro* conidial uptake by bone marrow neutrophils (BMNF) isolated from WT and MyD88^(−/−)^ mice was similar (S4C Fig. in S1 Text). These data indicate that the induction of neutrophil phagocytic and conidiacidal activities is not coupled to neutrophil-intrinsic MyD88 function during respiratory fungal infection.

### MyD88 is required for chemokine induction during *A. fumigatus* infection

**Figure 3:**
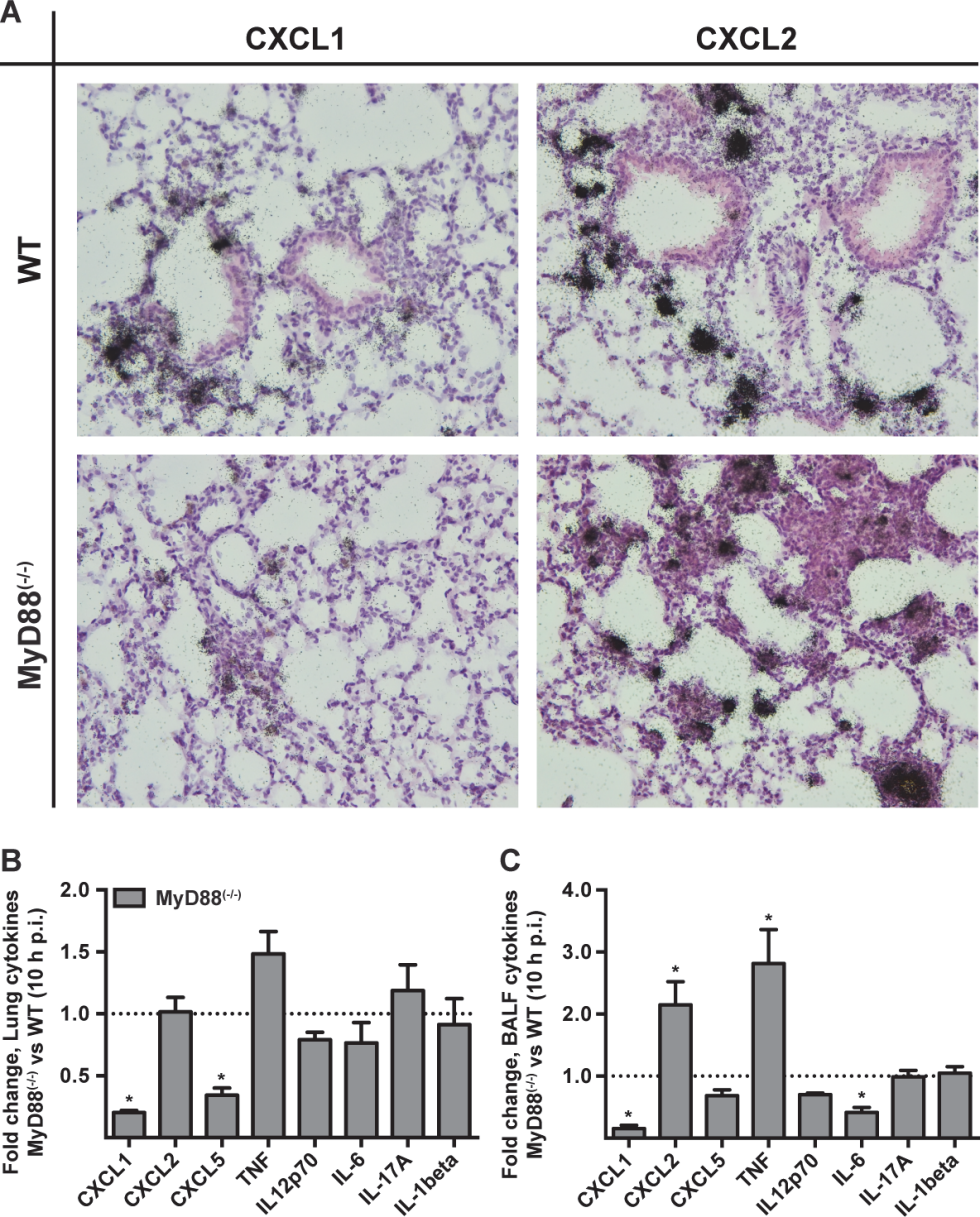
MyD88 is required for the first phase of CXCL1 induction in the lung. WT and MyD88^(−/−)^ mice were challenged with 3 × 10^7^ conidia and lung tissues were processed for (A) *in situ* mRNA hybridization or (B, C) chemokine analysis 10 h p.i. (A) The panels show representative WT (top row) and MyD88^(−/−)^ (bottom row) lung sections hybridized with ^35^S-labeled, CXCL1- (left column) and CXCL2-specific (right column) riboprobes and counterstained with hematoxylin. Representative micrographs from an experiment with 3 mice per group are shown at original magnification, 200×. (B) Lung or (C) BALF cytokines 10 h p.i. expressed as the fold change (+SEM) in the MyD88^(−/−)^ response compared to the WT response pooled from 2–3 experiments with 6–12 mice per genotype.

To investigate a link between the MyD88-dependent neutrophil recruitment defect and chemokine induction in the lung, we focused on the transcription and translation of the neutrophil-recruiting chemokines CXCL1/KC, CXCL2/MIP-2, and CXCL5/LIX since these chemoattractants act as agonists for the chemokine receptor CXCR2. Administration of anti-CXCR2 antibodies exacerbates mortality [8] and genetic deficiency in CXCR2 leads to delayed neutrophil recruitment following *A. fumigatus* challenge [7]. Because CXCL1 mRNA stability is regulated in part by inflammatory stimuli [36], we carried out *in situ* mRNA hybridization on lung sections of 3 × 10^7^ Af293 or PBS administered MyD88^(−/−)^ and WT mice, with gene-specific riboprobes for *cxcl1* and *cxcl2*. Representative high power micrographs from lung sections demonstrated a severely attenuated signal for *cxcl1* but not for *cxcl2* in infected MyD88^(−/−)^ mice compared to infected WT mice (Fig. 3A). No riboprobe signal was observed in uninfected controls (S5 Fig. in S1 Text). Although we could not unambiguously identify the cell types that produce CXCL1 and CXCL2 mRNA in infected mice, these data indicate that MyD88-dependent signals are required for CXCL1 but not for CXCL2 mRNA induction or stability in the lung following *A. fumigatus* challenge.

We next examined cytokine induction in *A. fumigatus*-infected WT and MyD88^(−/−)^ at the protein level by ELISA. The lung and BALF tissues in MyD88^(−/−)^ mice displayed a marked decrease in CXCL1 and CXCL5 levels, but not in CXCL2 levels at 10 h p.i. (Fig. 3B-3C, S6A-S6F Fig. in S1 Text) consistent with a prior study [29]. BALF and lung TNF were increased in MyD88^(−/−)^ mice. However, a number of other cytokines assayed (IL-12p70, IL-6, IL-17A, IL-1β) were not significantly reduced or elevated in BALF and lung tissues of MyD88^(−/−)^ mice at 10 h p.i. (Fig. 3B-3C). Thus, consistent with the MyD88-dependent defect in neutrophil recruitment, we observed a MyD88-dependent reduction of the chemokines CXCL1 and CXCL5 in the lung.

### Lung epithelial IL-1 receptor signaling drives MyD88-dependent neutrophil recruitment

**Figure 4:**
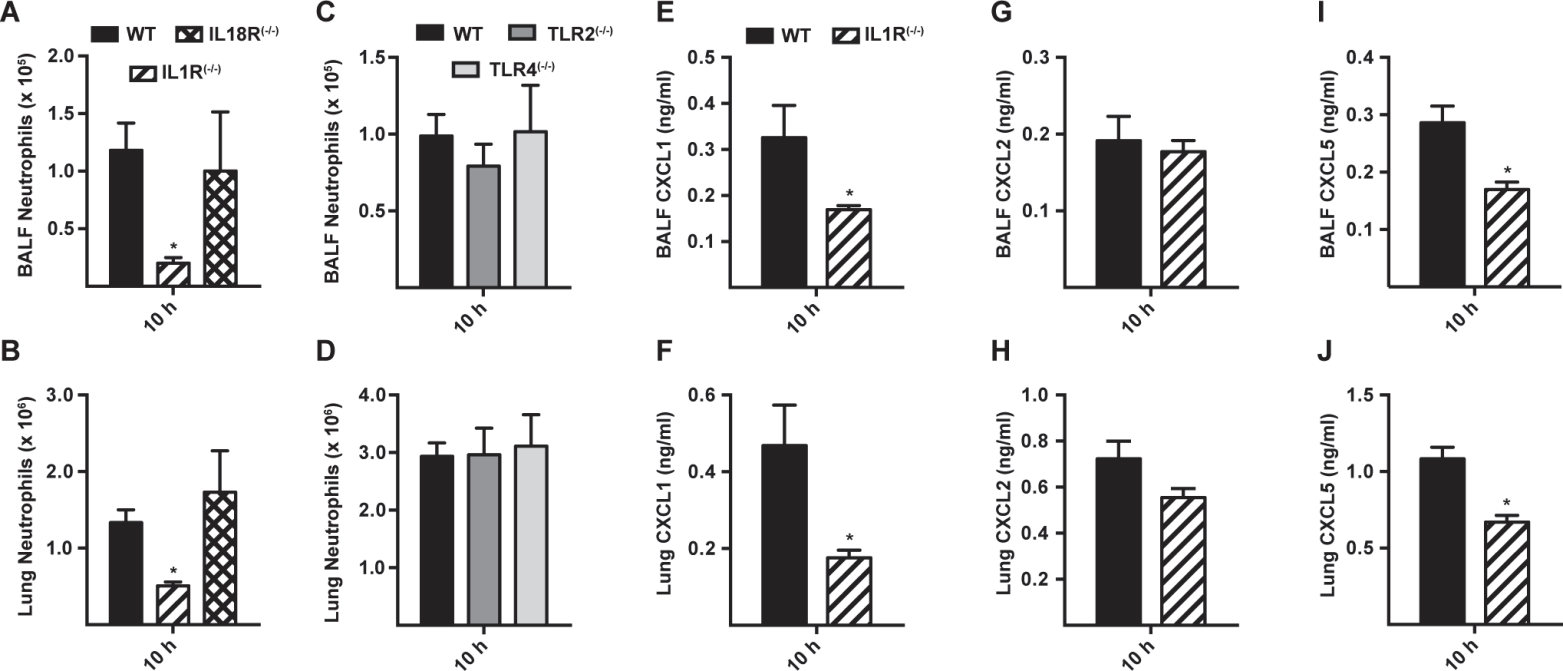
Interleukin-1 receptor signaling controls MyD88-dependent chemokine induction and neutrophil recruitment. Mean (+SEM) of (A, C) BALF, (B, D) lung neutrophil recruitment and (E, G, I) BALF and (F, H, J) lung, (E, F) CXCL1, (G, H) CXCL2 and (I, J) CXCL5 levels in WT (black bars), IL-1R^(−/−)^ (bars with diagonal stripes), IL-18R^(−/−)^ (bars with crosshatch), TLR2^(−/−)^ (dark grey bars), and TLR4^(−/−)^ (light grey bars) mice 10 h p.i. with 3 × 10^7^ conidia. Data are from 2 (A-D) or 1 (E-J) experiment(s) with 5–7 mice per genotype in each experiment. Graphs from a single experiment (out of three independent experiments) are shown for E, G and I.

Since MyD88 is critical for signal transduction from IL-1R family members and many TLRs, we focused on neutrophil recruitment and chemokine induction in IL-1R^(−/−)^, IL-18R^(−/−)^, TLR2^(−/−)^, and TLR4^(−/−)^ mice. Defective BALF and lung neutrophil recruitment was observed in IL-1R^(−/−)^ mice, but not in IL-18R^(−/−)^, TLR2^(−/−)^, and TLR4^(−/−)^ mice at 10 h p.i. (Fig. 4A-4D). In parallel, lung and BALF CXCL1 and CXCL5, but not CXCL2 levels were diminished in IL-1R^(−/−)^ mice at 10 h p.i. (Fig. 4E-4J), matching the results observed in MyD88^(−/−)^ mice (Figs. 2A-2B, 3B-3C, S6A-S6F in S1 Text). BALF CXCL1, CXCL2, and CXCL5 levels in TLR2^(−/−)^ and TLR4^(−/−)^ mice were not substantially different from those in WT mice at this time point (S7 Fig. in S1 Text).

At 36 h p.i., IL-1R^(−/−)^ mice displayed similar BALF neutrophil recruitment and CXCL1 and CXCL2 levels as in WT mice (S8A-S8C Fig. in S1 Text). BALF CXCL5 were decreased in IL-1R^(−/−)^ mice (S8D Fig. in S1 Text), similar to observations in MyD88^(−/−)^ mice at this time point (S6F Fig. in S1 Text). These data indicate that IL-1R signaling mediates the initial MyD88-dependent chemokine induction and neutrophil recruitment during respiratory *A. fumigatus* infection.

**Figure 5:**
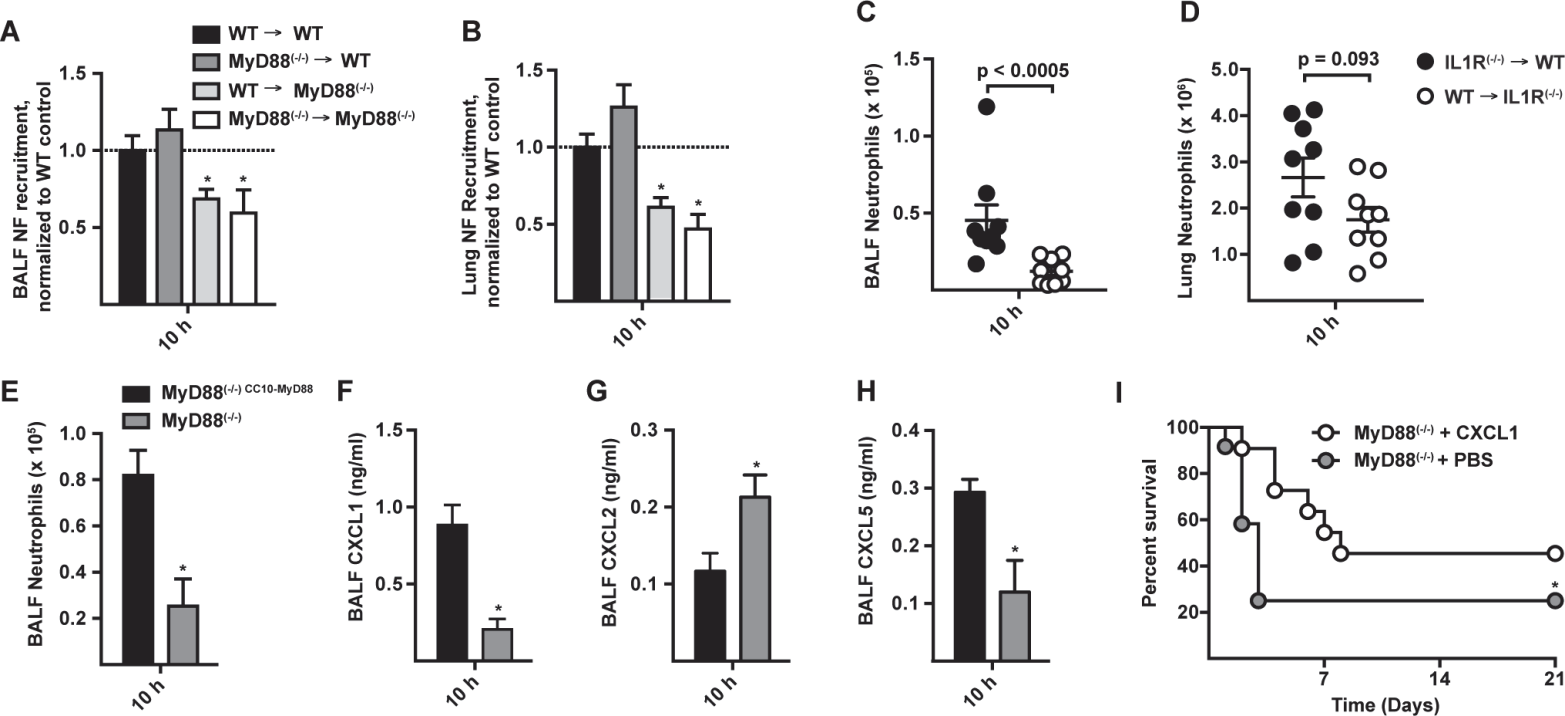
CXCL1 is controlled by MyD88 in lung epithelial cells and prolongs survival in MyD88^(−/−)^ mice following *A. fumigatus* challenge. (A) BALF (B) lung neutrophil recruitment in WT → WT (black bars), MyD88^(−/−)^ → WT (dark grey bars), WT → MyD88^(−/−)^ (light grey bars), and MyD88^(−/−)^ → MyD88^(−/−)^ (white bars) BM chimeric mice 10 h p.i. with 3 × 10^7^ conidia. Data are expressed as the fold change when compared to the WT → WT group and were pooled from 3 experiments with 12–15 mice per group. (C) BALF and (D) lung neutrophil recruitment in IL1R^(−/−)^ → WT (black circles), and WT → IL1R^(−/−)^ (white circles) BM chimeric mice 10 h p.i. with 3 × 10^7^ conidia. Data are expressed as mean (±SEM) and are from an experiment with 9 mice per group. (E-H) Mean (+SEM) BALF (E) neutrophil recruitment, (F) CXCL1, (G) CXCL2 and (H) CXCL5 levels, in MyD88^(−/−) CC10-MyD88^ (CC10-MyD88^+^; black bars) and in MyD88^(−/−)^ transgene-negative littermate controls (CC10-MyD88^−^; grey bars) 10 h p.i. with 3 × 10^7^ conidia. Data were pooled from 2 experiments and include 7–9 mice per genotype. (I) Kaplan-Meier survival plot of MyD88^(−/−)^ mice challenged with 6–7 × 10^7^ conidia and treated 4 h p.i. with 50 ng rCXCL1 (white circles, n = 11), or PBS vehicle (grey circles, n = 12). Data were pooled from 2 experiments (p = 0.026, Gehan-Breslow-Wilcoxon test).

To investigate the cellular compartment in which MyD88 signaling drives early neutrophil recruitment in our model, we generated bone marrow chimeric mice that lack MyD88 either in radioresistant or in radiosensitive cells. MyD88 expression in radioresistant cells (i.e. in WT → MyD88^(−/−)^ mice) was required for rapid neutrophil recruitment (Fig. 5A-5B), while MyD88 expression in radiosensitive hematopoietic cells (i.e. in MyD88^(−/−)^ → WT mice) was dispensable for this process. Consistent with this finding, IL-1R expression in radioresistant cells, but not in radiosensitive cells, was critical for airway neutrophil recruitment in *A. fumigatus*-infected BM chimeric mice (Fig. 5C-5D).

To examine the contribution of lung epithelial cells to chemokine induction and neutrophil recruitment, we analyzed MyD88^(−/−)^ mice that express MyD88 under control of the club cell 10 kDa protein (CC10) promoter, restricting MyD88 expression to lung epithelial cells [33,37]. CC10 promoter-driven MyD88 expression rescued neutrophil recruitment (Fig. 5E) and CXCL1 and CXCL5 induction (Fig. 5F-5H) in *A. fumigatus*-challenged mice compared to non-transgenic, globally MyD88-deficient littermate controls. These data indicate that early IL-1R/MyD88-dependent signals in lung epithelial cells recruit neutrophils to the infection site.

### Administration of rCXCL1 prolongs survival in MyD88^(−/−)^ mice

To determine whether ELR^+^ CXC chemokine induction and neutrophil recruitment represents the major mechanism by which MyD88 mediates rapid host defense against *A. fumigatus* conidia, we administered a single dose of recombinant CXCL1 (rCXCL1), CXCL2 (rCXCL2), CXCL5 (rCXCL5), or PBS diluent via the i.t. route to MyD88^(−/−)^ mice 4 h p.i. and monitored murine survival and neutrophil recruitment. The median survival of rCXCL1-treated mice was 8 days compared to 3 days for PBS-treated controls (Fig. 5I). The prolonged survival in rCXCL1-treated MyD88^(−/−)^ mice correlated with partial restoration of early neutrophil recruitment (S9 Fig. in S1 Text). In contrast, rCXCL2 or rCXCL5 did not prolong murine survival (S10 Fig. in S1 Text). Thus, murine mortality caused by a global deficiency in MyD88 signaling is partially reversed by administration of a single chemokine at a single time point, illustrating a central role of airway epithelial MyD88-dependent chemokine induction and neutrophil recruitment at the earliest stage of respiratory *A. fumigatus* infection.

### Hematopoietic CARD9 signaling drives a second phase of neutrophil recruitment

**Figure 6:**
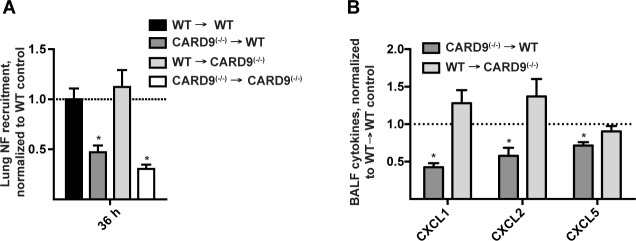
Hematopoietic CARD9 signaling drives neutrophil recruitment and chemokine induction. (A) Lung neutrophil recruitment in WT → WT (black bars), CARD9^(−/−)^ → WT (dark grey bars), WT → CARD9^(−/−)^ (light grey bars), and CARD9^(−/−)^ → CARD9^(−/−)^ (white bars) BM chimeric mice 36 h p.i. with 3 × 10^7^ conidia. (B) Chemokine levels in the CARD9^(−/−)^ → WT (dark grey bar), WT → CARD9^(−/−)^ (light grey bar) BM chimeric mice. Data are expressed as fold change when compared to the WT → WT control group and were pooled from 3 (A) or 2 (B) experiments with 4–5 mice per group in each experiment.

In a previous study, we found that CARD9 was dispensable for neutrophil recruitment and CXCL chemokine induction in the first 12 h p.i., However, our group and others found that Dectin-1^(−/−)^ and CARD9^(−/−)^ mice display defective chemokine production and neutrophil recruitment at 24 and 36 h p.i., respectively [14,23], time points that lie outside the MyD88-dependent period. We therefore investigated whether MyD88 and CARD9 act in the same or in different cellular compartments. By analyzing neutrophil recruitment in BM chimeric mice at 36 h p.i. we found that CARD9 deficiency in radiosensitive hematopoietic cells (i.e. CARD9^(−/−)^ → WT) leads to defective neutrophil airway and lung recruitment, similar to BM chimeric mice that are globally defective in CARD9 (i.e. CARD9^(−/−)^ → CARD9^(−/−)^) (Fig. 6A). Consistent with this finding, BALF CXCL1, CXCL2, and CXCL5 chemokine levels were reduced in CARD9^(−/−)^ → WT mice, but not in WT → CARD9^(−/−)^ mice (Fig. 6B), indicating that CARD9 acts in hematopoietic cells to mediate the second phase of ELR^+^ CXC chemokine induction and lung neutrophil recruitment. Thus, MyD88 and CARD9 act in different cellular compartments to regulate these processes.

### Neutrophils represent the principal cellular source of CXCL2 during the CARD9-dependent phase of recruitment

**Figure 7:**
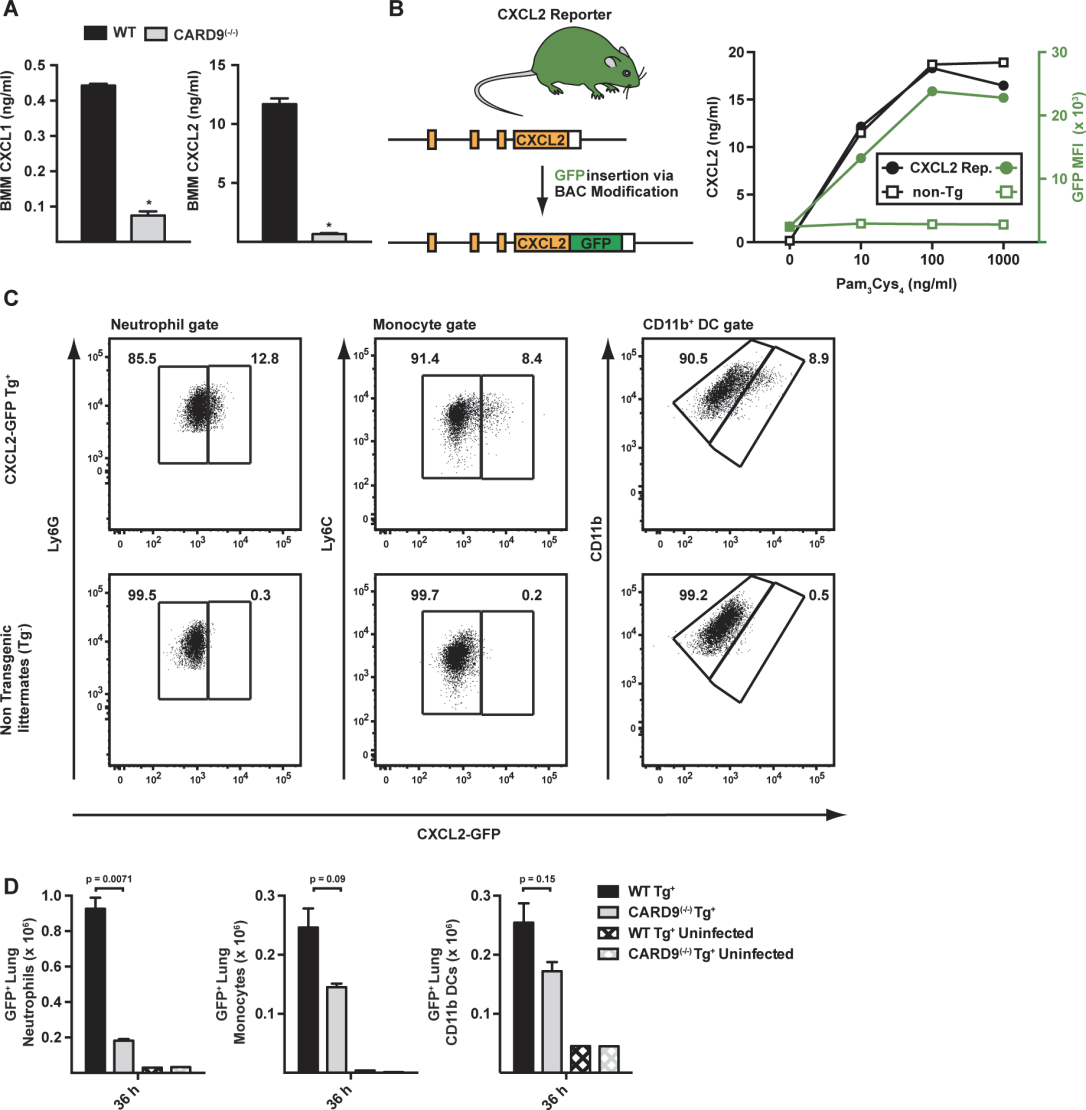
CARD9-dependent induction of ELR^+^ chemokines *in vitro* and *in vivo*. (A) The plots show mean (+SEM) CXCL1 and CXCL2 secretion by WT (black bars) or CARD9^(−/−)^ (light grey bars) BMMs following stimulation with *A. fumigatus* germlings (MOI = 1) as measured by ELISA. Data are from 4–5 replicates per condition from a representative experiment. (B) Strategy to generate CXCL2 reporter mouse. The graph shows CXCL2 (black lines) and mean GFP fluorescence (green lines) in transgene-positive (circle) and transgene-negative (non-Tg, square) littermates that were administered indicated amounts of Pam_3_Cys_4_ i.p. (C) The plots show neutrophils (left panel), inflammatory monocytes (middle panel) and CD11b^+^ DCs (right panel) that were isolated from CXCL2-GFP transgenic mice (upper panel) and non-transgenic littermates (lower panel) and analyzed for GFP expression. Representative data from 2 experiments is shown. Mice were administered 3 × 10^7^ conidia and lung cell suspensions were analyzed 36 h p.i. (D) The graphs show mean number (+SEM) of GFP^+^ lung neutrophils, inflammatory monocytes, or CD11b^+^ DCs from Tg^+^ CARD9^(+/+)^ (black bars), Tg^+^ CARD9^(−/−)^ (grey bars), Tg^−^ CARD9^(+/+)^ (black crosshatched bars) and Tg^−^ CARD9^(−/−)^ mice (grey crosshatched bars).

To determine whether fungal stimulation directly induces ELR^+^ CXC chemokine induction in hematopoietic cells, we cultured WT and CARD9^(−/−)^ BM macrophages (BMMs) with *A. fumigatus* germlings. BMMs produced CXCL1 and CXCL2 in a CARD9-dependent manner (Fig. 7A), though CXCL5 was not detected in these co-culture experiments.

To visualize the cellular source of CXCL2 in the murine lung, we generated a CXCL2 reporter mouse in which GFP expression was placed under control of the murine CXCL2 promoter (Fig. 7B). GFP transgene expression in BMMs prepared from CXCL2 reporter mice correlated with CXCL2 release triggered by TLR2 agonist stimulation (Fig. 7C). To define the cellular sources of CXCL2 and to examine CARD9-dependent regulation of CXCL2, we crossed to the transgene to the CARD9^(−/−)^ background and compared its expression in CARD9^(+/+)^ and CARD9^(−/−)^ mice infected with *A. fumigatus*. GFP expression was observed primarily in neutrophils, and to a lower frequency, in inflammatory monocytes and monocyte-derived CD11b^+^ DCs (Fig. 7D). In contrast, GFP-expressing CD3^+^, CD19^+^, or NK1.1^+^ cells were not observed in lungs of *A. fumigatus*-infected mice at 36 h p.i. Lung-infiltrating neutrophils thus represent the most abundant cellular source of CXCL2 and its expression was dependent on CARD9.

### MyD88 and CARD9 act sequentially to mount a protective inflammatory response against *A. fumigatus* infection

**Figure 8:**
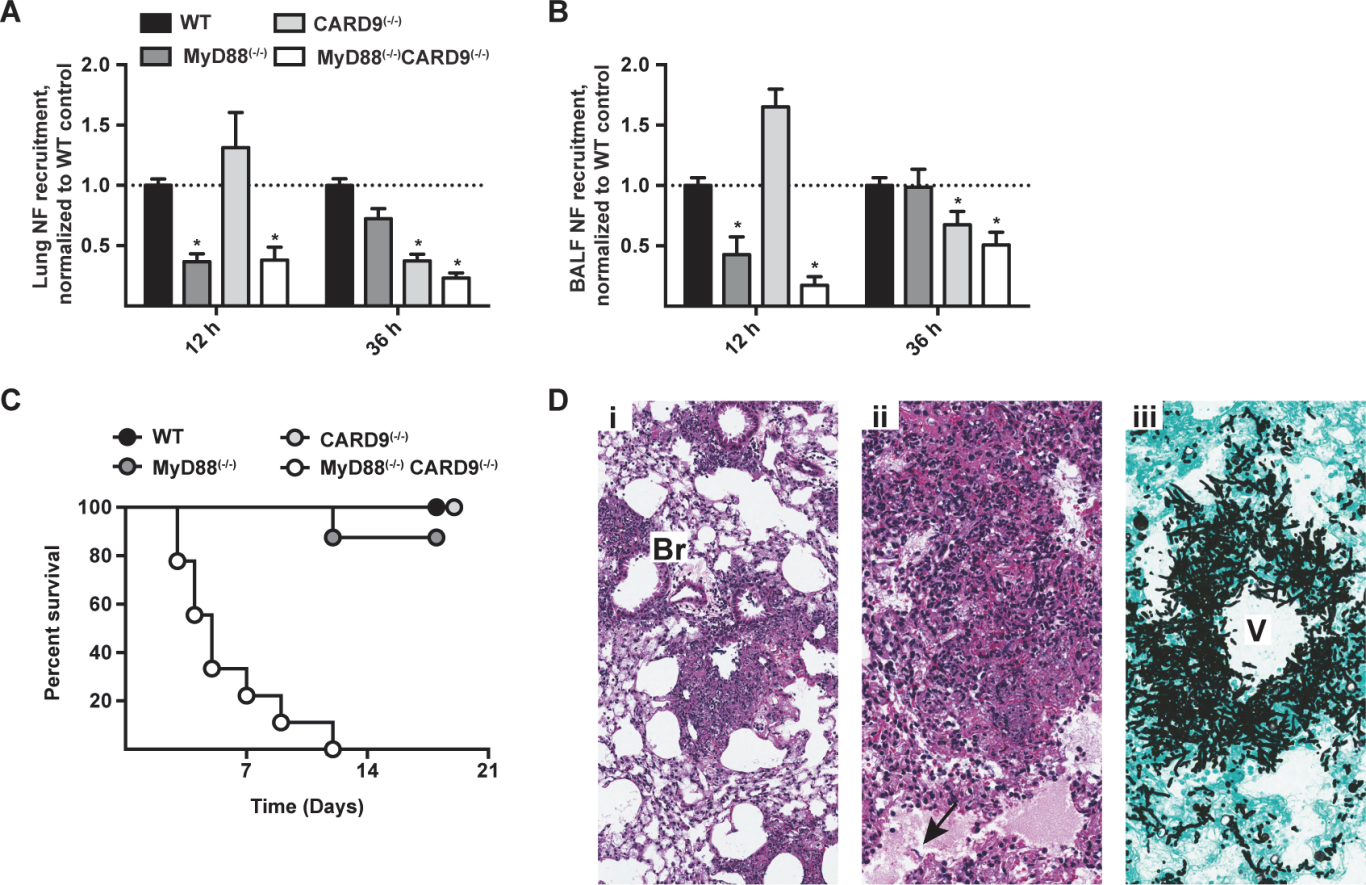
Effects of MyD88 and CARD9 are additive in murine defense against aspergillosis. (A-B) WT (black bars), MyD88^(−/−)^ (dark grey bars), CARD9^(−/−)^ (light grey bars), and MyD88^(−/−)^CARD9^(−/−)^ (white bars) mice were challenged with 3 × 10^7^ conidia and analyzed for neutrophil recruitment in (A) lung and (B) BALF at indicated time points. Data are pooled from 3 experiments with 10–15 mice per group and are expressed as the fold change when compared to the WT control group. To compare data on neutrophil recruitment from all four genotypes from multiple experiments, we pooled the experiments performed using WT, MyD88^(−/−)^ and MyD88^(−/−)^CARD9^(−/−)^ double knockout with our previously published results from WT, CARD9^(−/−)^ and Dectin-1^(−/−)^ experiments [23]. (C) Kaplan-Meier survival plot of WT (n = 9), MyD88^(−/−)^ (n = 8), CARD9^(−/−)^ (n = 6) and MyD88^(−/−)^CARD9^(−/−)^ (n = 9) mice infected with 3 × 10^7^ conidia. (D) Representative micrographs of H&E and GAS stained lung sections from MyD88^(−/−)^CARD9^(−/−)^ mice 48 h p.i. Images were captured at (Di) 20× and (Dii-Diii) 40× magnification. Arrow indicates region with alveolar edema and fungal hyphae. Br = bronchiole, V = vessel.

To define the temporal process by which MyD88- and CARD9-coupled signals mediate lung neutrophil recruitment, we compared these processes in MyD88^(−/−)^, CARD9^(−/−)^, MyD88^(−/−)^ CARD9^(−/−)^, and in C57BL/6 control mice. Consistent with a two-phase model of neutrophil recruitment, we observed MyD88-dependent, CARD9-independent neutrophil recruitment in the first phase and MyD88-independent, CARD9-dependent neutrophil recruitment in the second phase of respiratory fungal infection (Fig. 8A-8B).

Beyond its role in neutrophil recruitment, global CARD9 deficiency is associated with a defect in killing *A. fumigatus* [23] and *C. albicans* [38]. It is possible that CARD9 expression in neutrophils is essential for conidiacidal activity or, alternatively, that neutrophil-extrinsic CARD9 signaling regulates neutrophil conidiacidal activity. To distinguish these possibilities, we analyzed neutrophil conidial uptake and killing in mixed BM chimeric mice (CD45.2^+^ CARD9^(−/−)^ or CD45.2^+^ MyD88^(−/−)^CARD9^(−/−)^ BM cells mixed in a 1:1 ratio with CD45.1^+^ MyD88^(+/+)^CARD9^(+/+)^ BM cells and injected into lethally irradiated CD45.1^+^CD45.2^+^ MyD88^(+/+)^CARD9^(+/+)^ recipients) using FLARE conidia. In this experimental setting, CARD9^(−/−)^ and MyD88^(−/−)^CARD9^(−/−)^ neutrophils were not defective in conidial uptake or killing compared to WT (i.e. MyD88^(+/+)^CARD9^(+/+)^) counterparts isolated from the same lung (S11A - S11D Fig. in S1 Text). Thus, CARD9 signaling regulates neutrophil conidiacidal activity in a cell-extrinsic manner, since neutrophil-intrinsic CARD9 and MyD88 expression both appear dispensable for this process.

To examine the cumulative effect of MyD88 and CARD9 function in host defense, we challenged double knockout, single knockout, and control mice with a conidial dose (3 × 10^7^) that is sub-lethal for single adapter protein knockout strains. MyD88^(−/−)^CARD9^(−/−)^ mice rapidly and universally succumbed to this infectious inoculum, while the mice in all other groups survived the infection (Fig. 8C). Histopathological examination of hematoxylin and eosin (H&E) stained lung sections from MyD88^(−/−)^CARD9^(−/−)^ mice demonstrated severe disruption of pulmonary architecture and multifocal to coalescing areas of necrosis centered on and around vessels and bronchioles (Fig. 8Di-8Dii). Necrotic areas were characterized by abundant cellular and nuclear debris admixed with many fungal hyphae (Fig. 8Dii, arrow). GAS stained lung sections of MyD88^(−/−)^CARD9^(−/−)^ mice contained many germinating conidia and fungal hyphae expanding and surrounding vessels and bronchioles, indicative of severe tissue destruction associated with IA (Fig. 8Diii). Mice in other groups however, did not develop tissue-invasive hyphae during the course of study (S12 Fig. in S1 Text, arrows show regions of hyphal proliferation). Collectively, these data indicate a sequential and additive role for MyD88 and CARD9 signaling in host defense against *A. fumigatus*, with critical contributions from lung epithelial and hematopoietic cells in orchestrating chemokine and neutrophil recruitment to prevent tissue-invasive disease.

## Discussion

Rapid neutrophil influx is essential to prevent the germination and tissue invasion of airborne conidia that are inhaled daily during human life. *A. fumigatus* activates multiple innate immune signaling pathways that act in distinct cell types to induce an abundance of neutrophil chemotactic molecules. Deciphering the timing and importance of each component to the orchestration of effective innate immune defense has been challenging. In this study, we demonstrate that IL-1R/MyD88-mediated signals in lung epithelial cells and CARD9-mediated signals in hematopoietic cells provide sequential contributions to ELR^+^ CXC chemokine induction and to airway neutrophil recruitment. In the absence of either signaling pathway within the relevant cellular compartment, lung ELR^+^ CXC chemokine expression levels are diminished and neutrophil recruitment is either delayed at onset of fungal challenge (i.e. in MyD88 deficiency) or lags in the ensuing phase (i.e. in CARD9 deficiency). Among the ELR^+^ CXC chemokines, loss of MyD88 signaling was associated with defects in CXCL1 and to a lesser extent in CXCL5, while loss of CARD9 signaling was associated with defects in CXCL1 and CXCL2. Although this study and our previous work [23] demonstrate that loss in either signal adapter protein increases murine susceptibility to *A. fumigatus* challenge, host defense against inhaled conidia was profoundly compromised when both MyD88- and CARD9-mediated signals were absent. Neutrophil-intrinsic MyD88 and CARD9 function is dispensable for the induction of neutrophil phagocytic and conidiacidal activities, supporting the view that the primary role of both adaptors is to orchestrate neutrophil recruitment. This idea is reinforced by the observation that a single application of exogenous rCXCL1 prolongs survival in infected MyD88^(−/−)^ mice.

Given the essential requirement for CXCR2 ligands in host defense, our study did not focus on other neutrophil chemoattractants that can emerge in the vicinity of microorganisms, e.g. complement component C5a, or at endothelial sites, e.g. leukotriene B4 [39,40]. Other chemoattractants implicated in neutrophil chemotaxis include CCL3, CCL6, and CCL9 (via CCR1), platelet-activating factor (via 2 receptors), and CXCL12 (via CXCR4) [40]. Thus, it is possible that rCXCL1 administration enhances neutrophil recruitment by replenishing endogenous CXCL1 or by compensating for MyD88-dependent deficits in other chemoattractants that may act in series or parallel [39]. However, under the experimental conditions tested, rCXCL2 and rCXCL5 did not ameliorate MyD88-dependent mortality. One possible explanation is that endogenous CXCL2 and CXCL5 were relatively preserved in infected MyD88^(−/−)^ mice at 10 h p.i., unlike CXCL1, blunting the impact of exogenous administration. Jeyaseelan and colleagues reported that CXCL1 regulates expression of CXCL2 and CXCL5 during murine *Klebsiella pneumoniae* pneumonia [41]. During murine pulmonary *A. fumigatus* infection, the early defect in CXCL1, and to a lesser extent CXCL5, in MyD88^(−/−)^ mice was not associated with reduced CXCL2 levels, consistent with the notion that CXCL2 production in this model is not strictly dependent on CXCL1.

Our results indicate that lung epithelial cells play a central role in coordinating innate immune responses to inhaled conidia. We provide evidence for this statement in the form of experiments that demonstrate a critical role for MyD88 signaling in radioresistant cells, but not in radiosensitive cells, for CXCL1 and CXCL5 induction and neutrophil recruitment. In addition, expression of MyD88 under control of the well-characterized CC10 promoter [33] is sufficient to rescue CXCL1 and CXCL5 induction and neutrophil recruitment. A previous study on inflammatory responses to conidia in immortalized lung cell lines (BEAS-2B) implicated phosphatidylinositol-3-kinase, p38 MAP kinase, and ERK1/2 signaling pathways in the induction of interleukin-8 (IL-8) [42], a human equivalent of murine CXCL1. In that study, transfection of a dominant negative MyD88 mutant gene did not interfere with fungus-induced IL-8 release, though the findings were not extended to primary cells or to the lung tissue context. It is unclear whether co-culture of BEAS-2B cells and *A. fumigatus* induces IL-1R ligands *in vitro*. This step however, appears to be critical for epithelial cell activation in the lung and ensuing neutrophil airway trafficking following respiratory *A. fumigatus* challenge. This view is further supported by neutrophil recruitment studies in the lung following challenge with common bacterial agents of pneumonia including *Legionella pneumophila* [32,34], *Pseudomonas aeruginosa* [33], and *Streptococcus pneumonia* [43]. Additionally, in a cutaneous *Staphylococcus aureus* infection model, IL1R-MyD88 signaling mediates neutrophil recruitment in a TLR-independent manner [44,45].

How does *A. fumigatus* trigger IL-1R signaling in lung epithelial cells? Within 2 hours following intratracheal (i.t.) *A. fumigatus* challenge, alveolar macrophages transcribe *Il1α* and *Il1β* genes [46], and bioactive IL-1α and IL-1β is detectable in the lungs at 6 hours p.i. (Caffrey A. et al., manuscript submitted) coincident with the onset of a neutrophilic cellular influx [7]. In a parallel study, Obar and colleagues unveil a dominant role for IL-1α in early lung CXCL1 chemokine induction and neutrophil recruitment (Caffrey A. et al., manuscript submitted). Lung-resident myeloid cells represent a potential source of IL-1α and IL-1β at the earliest time point post-infection. *A. fumigatus* infection activates the transcription factor hypoxia-inducible factor-1α (HIF1α) in myeloid cells and this process is linked to defective IL-1α and CXCL1 induction, impaired neutrophil recruitment, and murine susceptibility to IA [47]. Epithelial cells may contribute to early IL-1α release as well, particularly in the context of tissue damage or injury, since pro-IL-1α constitutively expressed in lung epithelial cells, and upon necroptotic cell death, is processed via calpain-dependent cleavage and released [48,49]. IL-1α can rapidly induce chemotactic mediators and neutrophil recruitment to sites of inflammation, as demonstrated in sterile models of ischemic and hypoxic injury [48,49]. This model of IL-1α function is expanding to include microbial agents of inflammation, e.g. *L. pneumophila* within the respiratory tract [34]. Since respiratory fungal infection induces hypoxic tissue environments in the lung [50], receptor-interacting protein kinase 3 (RIP3)-dependent induction of necroptotic cell death [51] by inhaled conidia may provide a stimulus for IL-1α release. Although the impact of *A. fumigatus* conidia on necroptotic cell death—which favors IL-1α release—has not been investigated in detail, conidial melanin appears to inhibit apoptotic cell death—which favors IL-1β release—in macrophages and in epithelial cells *in vitro* [52–55]. Thus, the presence of conidial melanin and modulation of host cell death pathways may shape early IL-1α/β responses during respiratory fungal infection.

MyD88 acts as a transducer not only for IL-1R-mediated signals, but also for other IL-1R family members [48]. Previous studies indicate that *A. fumigatus* induces the synthesis of IL-1 receptor antagonist (IL-1RA) and the transcription of IL-36γ and IL-36 receptor antagonist in human peripheral blood mononuclear cells [56,57]. Although our studies and those of Obar and colleagues demonstrate a critical role for IL-1R/MyD88 signaling in host defense against *A. fumigatus*, our findings do not exclude a role for other IL-1R family members, e.g. IL-36R, in linking IL-1R-dependent signals to ELR^+^ CXC chemokine induction.

Our data did not reveal a requirement for CARD9 signaling in IL-1β and CXCL1, CXCL2, and CXCL5 induction and in airway neutrophil recruitment within the first 10 h p.i. In contrast, we and other investigators reported Dectin-1- and CARD9-dependent defects in IL-1α/β and CXCL1 and CXCL2 production at later time points, starting at 24 h post-infection [14,23]. In response to fungal stimuli, Dectin-1/Syk/CARD9 signaling is associated with activation of canonical NLRP3/ASC/caspase-1 inflammasomes [13,24] and of non-canonical ASC/caspase-8-containing inflammasomes [25]; both mediate the conversion of pro-IL-1β to biologically active IL-1β. Obar and colleagues observe that genetic deletion of ASC, critical for assembly of caspase-1- and caspase-8-containing inflammasomes does not diminish lung neutrophil recruitment nor increase susceptibility to IA, in contrast to IL-1R deficiency (Caffrey A. et al., manuscript submitted). Thus, CARD9-dependent production of IL-1β does not appear to be the primary trigger for IL-1R signaling, CXCL1 induction, and neutrophil recruitment in the initial phase of infection, though CLR/CARD9-dependent signals play a significant role in amplifying and sustaining neutrophil recruitment at later time points.

In the respiratory fungal infection model, MyD88-dependent CXCL1 and CXCL5 release correlates with early phase lung and airway neutrophil recruitment. Remarkably, administration of recombinant CXCL1 ameliorates murine mortality in MyD88^(−/−)^ mice, suggesting that MyD88-dependent control over neutrophil chemotaxis represents the major physiologically relevant function of early IL-1R/MyD88 signaling. In addition, MyD88 signaling negatively regulates TNF induction in *A. fumigatus*-infected mice, as demonstrated by our study and others [29]. Thus, TNF-induced lung tissue damage and necrosis, associated with defective ELR+ CXC chemokine induction, defective neutrophil recruitment, and defective fungal clearance, likely contributes to observed infectious outcomes.

In our study, CARD9 regulates CXCL1 and CXCL2 release in the second phase of neutrophil recruitment, with neutrophils acting as the primary hematopoietic source of CXCL2. Several explanations are possible for the differential temporal regulation and roles of CXCR2 ligands during pulmonary aspergillosis. First, lung and airway CXCL1 levels increase more rapidly than CXCL2 levels at the onset respiratory fungal infection [23,29] and thus CXCR2-dependent trafficking steps may reflect the relative abundance of cognate ligands. Second, since different resident and recruited cell types (e.g. neutrophils for CXCL2) may represent the major sources of these chemoattractants, the arrival and position of cellular CXCL1 or CXCL2 producers during neutrophil lung influx may determine the relative timing and role of CXCL1 and CXCL2 during this process. In addition, the relative access of individual ELR^+^ chemokines to glycosaminoglycans during fungal challenge is likely to play a key role in forming gradients and directing neutrophil trafficking into the lung [58,59]. To address these issues, the development of a complete set of transgenic reporters and conditional ablation strategies for CXCR2 ligands will facilitate more incisive experiments to determine the relative contribution, anatomic localization, cellular sources, and regulation of individual CXCR2 ligands during respiratory fungal infection.

In humans, Mendelian defects in MyD88 and CARD9 signaling do not lead to the spontaneous development of aspergillosis, unlike individuals with Mendelian defects in NADPH oxidase activity (i.e. chronic granulomatous disease). Children that lack MyD88 signaling develop pyogenic infections, primarily due to *Streptococcus pneumonia* [60], and individuals with CARD9 deficiency develop mucocutaneous and disseminated candidiasis and dermatophytosis [38,61,62]. These observations support the model that IL-1R/MyD88 and CLR/CARD9 signaling provide redundancy in orchestrating sterilizing immunity against inhaled conidia. Since humans typically inhale several hundred to several thousand conidia daily [63], the presence of a single functional adapter pathway is sufficient to mediate fungal clearance in an otherwise immune competent host. Consistent with this idea, we observe that genetic deletion of both adapter proteins significantly increases murine susceptibility to invasive disease compared to deletion of a single adapter protein. In the context of allogeneic hematopoietic cell transplantation (HCT), genetic variations in the IL-1 gene cluster are associated with susceptibility to IA, though these data must be interpreted cautiously since an independent validation of this cohort has not been reported [64]. Allelic differences in components of CLR/CARD9 and in MyD88 signaling pathways have been linked to susceptibility to IA in allogeneic HCT patients in multiple cohorts [12,65–68]. Thus, genetic variants in CARD9- or in MyD88-dependent signaling pathways may predispose to IA only in the setting of innate immune damage (e.g. in patients with neutropenia or in patients that receive corticosteroids).

In sum, our study reveals coordinated regulation of chemokine induction and neutrophil recruitment by IL-1R/MyD88 and CLR/CARD9 signaling pathways that operate in a biphasic manner and in epithelial and hematopoietic compartments to orchestrate sterilizing immunity against *A. fumigatus*. Further insight into the signals and temporal sequence of events required for innate immune orchestration is likely to inform strategies to identify and intervene in patients at high risk for aspergillosis.

## Materials and Methods

### Ethics statement

All animal experiments were conducted with sex- and age-matched mice and performed with approval from the FHCRC (protocol number 1813) and MSKCC (protocol number 13-07-008) Institutional Animal Care and Use Committee. Animal studies were compliant with all applicable provisions established by the Animal Welfare Act and the Public Health Services Policy on the Humane Care and Use of Laboratory Animals.

### Reagents and antibodies

Chemicals and cell culture reagents were purchased from Sigma-Aldrich and Gibco respectively. *A. fumigatus* strain Af293 was used for all experiments.

### Mice

MyD88^(−/−)^ (Stock No. 009088, Jackson Laboratories) and CARD9^(−/−)^ [69] were crossed to generate MyD88^(−/−)^CARD9^(−/−)^ mice. IL-1R^(−/−)^ (Stock No. 003245), IL-18R^(−/−)^ (Stock Number 004131), C57BL/6 (CD45.2^+^), C57BL/6.SJL (CD45.1^+^), mice were from Jackson Laboratories. TLR2^(−/−)^ and TLR4^(−/−)^ mice were obtained by the Pamer laboratory from the Medzhitov laboratory in 2000 and backcrossed at least 10 generations on the C57BL/6 background. All mouse strains were bred and housed in the FHCRC Comparative Medicine Shared Resources or in the MSKCC Research Animal Resource Center under specific pathogen-free conditions. MyD88^(−/−)CC10-MyD88^ [33] and non-transgenic MyD88^(−/−)^ littermate controls were bred and maintained at the Yale University Animal Resources Center and shipped to MSKCC for experimental use.

### Generation of CXCL2 reporter mice

CXCL2 reporter mice were generated by bacterial artificial chromosome (BAC)-mediated transgenesis using the recombineering strategy developed by Heintz and colleagues [70]. The endogenous CXCL2 locus on BAC clone RP23-87C13 (BACPAC Resources Center, Children’s Hospital Oakland research Institute) was modified 3’ to the terminal Asn residue in the fourth exon to encode the following insertion in the 5’ to 3’ direction: an HA peptide (-YPYDVPDY-), a -GSG- linker, a 2A peptide (-GSGAPVKQTLNFDLLKLAGDVESNPGP-) and amino acids 1–238 of enhanced GFP followed by a stop codon. The BAC modification procedures were performed as described in [71,72] and the modified BAC was analyzed by DNA sequencing and Southern blotting to verify GFP integration at the CXCL2 locus. The purified modified RP23-87C13 BAC was injected into fertilized B6/129 oocytes at the University of Washington Transgenic Resources Program. Four potential founder animals were identified among offspring screened by PCR. One candidate, designated 3090, transmitted the CXCL2-GFP transgene to one-half of the progeny and was the founder of the CXCL2 reporter colony. CXCL2 reporter mice were backcrossed 10 generations to C57BL/6 mice. To generate CXCL2 reporter mice on the CARD9^(−/−)^ background, N8 CXCL2 reporter mice were crossed to CARD9^(−/−)^ mice and littermate non-transgenic CARD9^(−/−)^ controls were used in experiments.

### Generation of BM chimeric mice

BM chimeric mice were generated by reconstituting lethally irradiated (9.5 Gy) recipients (C57BL/6.SJL, CD45.1^+^CD45.2^+^ C57BL/6, MyD88^(−/−)^, or CARD9^(−/−)^ mice ) mice with 2–5 × 10^6^ MyD88^(−/−)^, CARD9^(−/−)^, MyD88^(−/−)^CARD9^(−/−)^ or C57BL/6 BM cells. The mice were treated with enrofloxacin or amoxicillin-clavulanate in the drinking water for 14 days to prevent bacterial infections and rested for 6–8 weeks prior to use in experiments.

### Analysis of infected mice

BAL and lung suspensions were prepared for flow cytometry as described in [72]. Briefly, lung digest and, if applicable, BAL cells were enumerated and stained with the following Abs: anti-Ly6C (clone AL-21), anti-Ly6G (clone 1A8), anti-CD11b (clone M1/70), anti-CD11c (clone HL3), anti-CD45.1 (clone A20), anti-CD45.2 (clone 104), anti-Ly6B.2 (clone 7/4), anti-MHC class II (clone M5/114.15.2). Neutrophils were identified as CD45^+^CD11b^+^Ly6C^lo^Ly6G^+^Ly6B.2^+^, monocytes as CD45^+^CD11b^+^Ly6C^hi^Ly6G^−^Ly6B.2^+^, lung macrophages as autofluorescent CD45^+^CD11c^+^MHC class II variable population, CD11b DCs as CD45^+^CD11c^+^MHC class II variable CD11b^+^ population and alveolar macrophages as SSC^hi^CD45^+^CD11b^+^CD11c^+^ BALF cells. Flow cytometry data was collected on a BD LSR II flow cytometer and analyzed on FlowJo, version 9.7.6 (Treestar).

Perfused murine lungs were homogenized in 2 mL of PBS, 0.025% Tween-20 for colony-forming units (CFUs) and for ELISA. For histology, perfused lungs were fixed in 10% neutral-buffered formalin, embedded in paraffin, sectioned at 4μm and stained with hematoxylin & eosin (H&E) or Gomori’s ammoniacal silver (GAS). Slides were reviewed by a board certified pathologist (SEK). Images were captured from whole slide images, acquired with the Aperio ScanScope (Aperio Technologies) using ×20 and ×40 objectives at the FHCRC’s Experimental Histopathology Shared Resource. Images for S12 Fig. were captured using a Zeiss Mirax Midi slide scanner with 20×/0.8NA objective and analyzed using Pannoramic Viewer (v1.15.3) at the MSKCC’s Molecular Cytology Core Facility (MCCF).

To assess fungal burden, infected mice were euthanized and lungs were immediately frozen in liquid nitrogen. Samples were freeze-dried, homogenized with glass beads on a Mini Beadbeater (BioSpec Products, Inc., Bartlesville, OK), and DNA extracted with E.N.Z.A fungal DNA kit (Omega Bio-Tek, Norcross, GA) or phenol chloroform extraction. RT-PCR was performed as described previously [73]. LDH (CytoTox96 non-radioactive cytotoxicity assay kit; Promega, Cat. No. G1780) and albumin assays (Albumin (BCG) reagent set; Eagle Diagnostics, Cat. No. 2050-1) were performed on BALF collected from infected mice using the manufacturers’ protocols as follows. 100 μl of BALF was added to equal volumes of the recommended reagents and incubated for either 30 min (LDH) or 5 min (Albumin) and absorbance measured at 490 nm (LDH assay) or 630 nm (Albumin assay).

For *in situ* hybridization experiments, gene-specific riboprobes were synthesized by *in vitro* transcription using a Maxiscript SP6/T7 kit (Ambion) and unincorporated nucleotides were removed using RNA Mini Quick Spin columns (Roche). Paraffin embedded lung sections were pretreated as described [74], following deparaffinization in xylenes and rinsing in ethanol. *In situ* hybridization with ^35^S-UTP-labeled riboprobes (antisense or control sense) was performed as described previously [75] with 0.1M dithiothreitol included in the hybridization mix. Hybridizations were performed at 50°C overnight. After stringent washing to remove unbound riboprobes, tissue sections were coated with NTB emulsion (Kodak) and exposed at 10°C for 14 days. Parallel hybridizations using the control sense riboprobes did not give rise to specific autoradiographic signals.

For supplementation experiments, infected mice were administered 50 ng of recombinant rCXCL1 (Cat. No. 573702, BioLegend), rCXCL2 (Cat. No. 582502) or rCXCL5 (Cat. No. 573302) in 50 μl PBS or PBS alone via the i.t. route (at 4 h p.i.) and monitored for survival or assayed for neutrophil recruitment. 50 ng of recombinant rCXCL1 was analyzed using the Limulus amoebocyte lysate assay kit (Lonza, Cat. No. 50647U) and endotoxin content was below the level of detection (0.1 EU/ml).

### *In vitro Aspergillus*-BMM co-culture assays

BMM stimulation with *A. fumigatus* germlings was performed as described in [23]. Briefly, BMMs derived from WT and CARD9^(−/−)^ mice were stimulated with UV-inactivated germlings (MOI = 1) for 18 h. Chemokine levels in culture supernatants were measured using ELISA kits from R&D Systems.

### Statistical analysis

All results are expressed as mean (±SEM) derived from 3 independent experiments, unless stated otherwise. A Mann-Whitney U test was used for unpaired two groups or Wilcoxon signed-rank test for paired two groups comparison. Non-parametric Kruskal-Wallis one-way analysis of variance followed by Dunn’s post test was used for three groups comparison. Survival data was analyzed by long rank test unless stated otherwise. All statistical analyses were performed with GraphPad Prism software, v6.0c. A p value < 0.05 was considered significant and indicated with an asterisk.

## Supporting Information

S1 Text Supplementary figures and figure legends. (DOCX)

S2 Text Supplementary Materials and Methods. Generation of fluorescent *Aspergillus* reporter (FLARE) conidia. *In vitro* Neutrophil Assays. Calculation of Fungal Uptake and Viability in Leukocytes.
(DOCX)
