# Supplementary material for: Compartment-Specific and Sequential Role of MyD88 and CARD9 in Chemokine Induction and Innate Defense during Respiratory Fungal Infection
Source: PLoS Pathog. 2015 Jan 26;11(1):e1004589. doi: 10.1371/journal.ppat.1004589 (PMC4306481; doi:10.1371/journal.ppat.1004589)
Supplement: S1 Text — (DOCX) [file ppat.1004589.s001.docx]

**Text S1**

**Supplemental Figure Legends**

**Figure S1 (Supplementary to Figure 1). Histopathology of MyD88^(-/-)^ mice following *A. fumigatus* challenge.**

Representative micrographs of H&E and GAS stained lung sections from WT and MyD88^(-/-)^ mice 48 h p.i. (7 x 10^7^ conidia) are shown (A-B) at 2.5x or (C-D) 40x magnification. The white dashed line in (D) delineates a region of severe tissue necrosis which was commonly observed in MyD88^(-/-)^ mice. Br = bronchiole, V = vessel. Data are representative from two independent experiments with 8-10 mice per group.

**Figure S2 (Supplementary to Figure 2). MyD88 and lung myeloid cell populations following *A. fumigatus* challenge.**

(A-D) The bar graphs show the mean (+SEM) numbers of (A) lung inflammatory monocytes (B) lung macrophages (C) alveolar macrophages and (D) CD11b^+^ DCs numbers at 10 h and 36 h p.i. in C57BL/6 (black bars) and MyD88^(-/-)^ (grey bars) mice challenged with 3 x 10^7^ conidia. Data are pooled from 3 independent experiments and include 11-14 mice per genotype. (E-G) Flow plots of stained (E-F) lung and (G) BALF cell suspensions depict the gating strategies for leukocyte populations analyzed in this study.

**Figure S3 (Supplementary to Figure 2). Global MyD88 deficiency and neutrophil anticonidial effector functions.**

WT (black circles) and MyD88^(-/-)^ (grey circles) mice were infected with 3 x 10^7^ FLARE conidia and euthanized 10 h or 36 h p.i. (A) The plots are gated on CD45^+^CD11b^+^Ly6G^+^ lung neutrophils and analyzed for dsRed and Alexa Fluor 633 fluorescence. The tan gates indicate bystander neutrophils, and the red (R1) and blue (R2) gates indicate neutrophils that contain live or killed conidia, respectively, as demonstrated in [[1](#_ENREF_1)]. (B-C) The graphs show (B) neutrophil conidial uptake (R1 + R2) and (C) neutrophil conidial viability (R1/(R1+R2)) (±SEM) from a representative experiment with 3-4 mice per genotype and time point. (D-E) The graphs show neutrophil conidial uptake and neutrophil conidial viability using normalized data pooled from 3 experiments, each with 3-5 mice per genotype and time point.

**Figure S4 (Supplementary to Figure 2). Neutrophil-intrinsic MyD88 is dispensable for conidial uptake and killing.**

(A-B) BM chimeric mice (CD45.1^+^ MyD88^(+/+)^ and CD45.2^+^ MyD88^(-/-)^ BM cells → irradiated CD45.1^+^CD45.2^+^ MyD88^(+/+)^ recipients) were challenged with 3 x 10^7^ FLARE conidia. The scatterplots show the average (±SEM) frequency of (A) lung neutrophil conidial uptake by or (B) lung neutrophil conidial viability in CD45.1^+^ MyD88^(+/+)^ and CD45.2^+^ MyD88^(-/-)^ neutrophils at 10 h p.i. The data were analyzed as outlined in **Figure S3A-S3C**. Data are representative of 2 independent experiments with 4-5 mice per genotype. (C) The scatter plot shows BM neutrophil fungal uptake (±SEM) by WT and MyD88^(-/-)^ BM neutrophils at a MOI of 1 after a 16 h co-incubation at 37^o^C. One of 2 experiments is shown.

**Figure S5 (Supplementary to Figure 3). ISH analysis to detect the basal levels of chemokines in WT and MyD88^(-/-)^ mice lung.**

Lungs from PBS-treated WT and MyD88^(-/-)^ mice (uninfected controls) were processed for *in situ* mRNA hybridization to detect basal levels of CXCL1 and CXCL2 mRNA. Representative micrographs of WT (top row) and MyD88^(-/-)^ (bottom row) lung sections, hybridized with ^35^S-labeled, CXCL1- (left column) and CXCL2-specific (right column) riboprobes and counterstained with hematoxylin, are shown at original magnification, 200x.

**Figure S6 (Supplementary to Figure 3). MyD88 is required for rapid CXCL1 and CXCL5 induction during *A. fumigatus* challenge.**

(A-F) The plots show mean (±SEM) (A, D) CXCL1, (B, E) CXCL2 and (C, F) CXCL5 levels in the (A-C) lung and (D-F) BALF of WT (black circles) and MyD88^(-/-)^ (grey circles) mice challenged with 3 x 10^7^ conidia and euthanized at the indicated time points. Data are from 1-2 (Lung) or 2-3 (BALF) experiments with 6-12 mice per time point and genotype.

**Figure S7 (Supplementary to Figure 4). BALF chemokine profiles in WT, TLR2^(-/-)^, and TLR4^(-/-)^ mice.**

(A-C) BALF CXCL1, CXCL2 and CXCL5 levels in the WT (black bars), TLR2^(-/-)^ (dark grey bars) and TLR4^(-/-)^ (light grey bars) mice at 10 h p.i. with 3 x 10^7^ conidia. Data are expressed as mean (±SEM) and pooled from two experiments with 4-5 mice per genotype in each experiment.

**Figure S8 (Supplementary to Figure 4). Neutrophil recruitment and chemokine levels in IL1R^(-/-)^ mice at 36 h p.i.**

The plots show (A) airway neutrophil recruitment and (B-C) BALF chemokine levels in infected WT and IL1R^(-/-)^ mice at 36 h p.i with 3 x 10^7^ conidia. Data are expressed as mean (+SEM) and are from an experiment with 4-5 mice per genotype.

**Figure S9 (Supplementary to Figure 5). rCXCL1 increases lung neutrophil recruitment in *A. fumigatus*-challenged MyD88^(-/-)^ mice.**

MyD88^(-/-)^ mice challenged with 3 x 10^7^ conidia were treated with 50 ng rCXCL1 (white bars) or 50 ng rCXCL2 (grey bars) or PBS diluent (black bars) via the i.t. route at 4 h p.i. Neutrophil recruitment was analyzed at the infection site 10 h p.i. Data are shown as mean (+SEM) from an experiment with 5 mice per group.

**Figure S10 (Supplementary to Figure 5). Chemokine supplementation and survival analysis in MyD88^(-/-)^ mice.**

Kaplan-Meier survival plot of MyD88^(-/-)^ mice challenged with 6 x 10^7^ conidia and treated 4 h p.i. with 50 ng rCXCL2 (white triangles, n = 10) or 50 ng rCXCL5 (white hexagons, n = 10) or PBS vehicle (grey circles, n = 10).

**Figure S11 (Supplementary to Figure 8). Neutrophil-intrinsic MyD88 and CARD9 are dispensable for conidial uptake and killing in the lung.**

(A-D) A 1:1 mix of (A-B) CD45.1^+^ CARD9^(+/+)^ and CD45.2^+^ CARD9^(-/-)^ BM cells or (C-D) CD45.1^+^ MyD88^(+/+)^CARD9^(+/+)^ and CD45.2^+^ MyD88^(-/-)^CARD9^(-/-)^ BM cells (C-D) were infused into lethally irradiated CD45.1^+^CD45.2^+^ recipients. BM chimeric mice were rested for 6 weeks and challenged with 3 x 10^7^ FLARE conidia. The scatter plots show the average (±SEM) (A and C) lung neutrophil conidial uptake and (B and D) lung neutrophil conidial viability at 10 h p.i. for cells of the indicated genotype. The data were evaluated as outlined in Figure S3A-S3C. Data are representative of 2 independent experiments with 3-5 mice per genotype.

**Figure S12 (Supplementary to Figure 8). MyD88^(-/-)^CARD9^(-/-)^ mice display extensive hyphal proliferation during conidial challenge.**

Representative micrographs of GAS stained lung sections from WT, MyD88^(-/-)^, CARD9^(-/-)^ and MyD88^(-/-)^CARD9^(-/-)^ mice 2-3 days p.i. (7 x 10^7^ conidia) are shown at 2x magnification. Data are representative from two independent experiments with 8-10 mice per genotype. Notice the extensive hyphal growth in MyD88^(-/-)^CARD9^(-/-)^group as indicated by arrows.

**Supplemental References**

1. Jhingran A, Mar KB, Kumasaka DK, Knoblaugh SE, Ngo LY, et al. (2012) Tracing conidial fate and measuring host cell antifungal activity using a reporter of microbial viability in the lung. Cell Rep 2: 1762-1773.

**Figure S1 (Supplementary to Figure 1)**

**
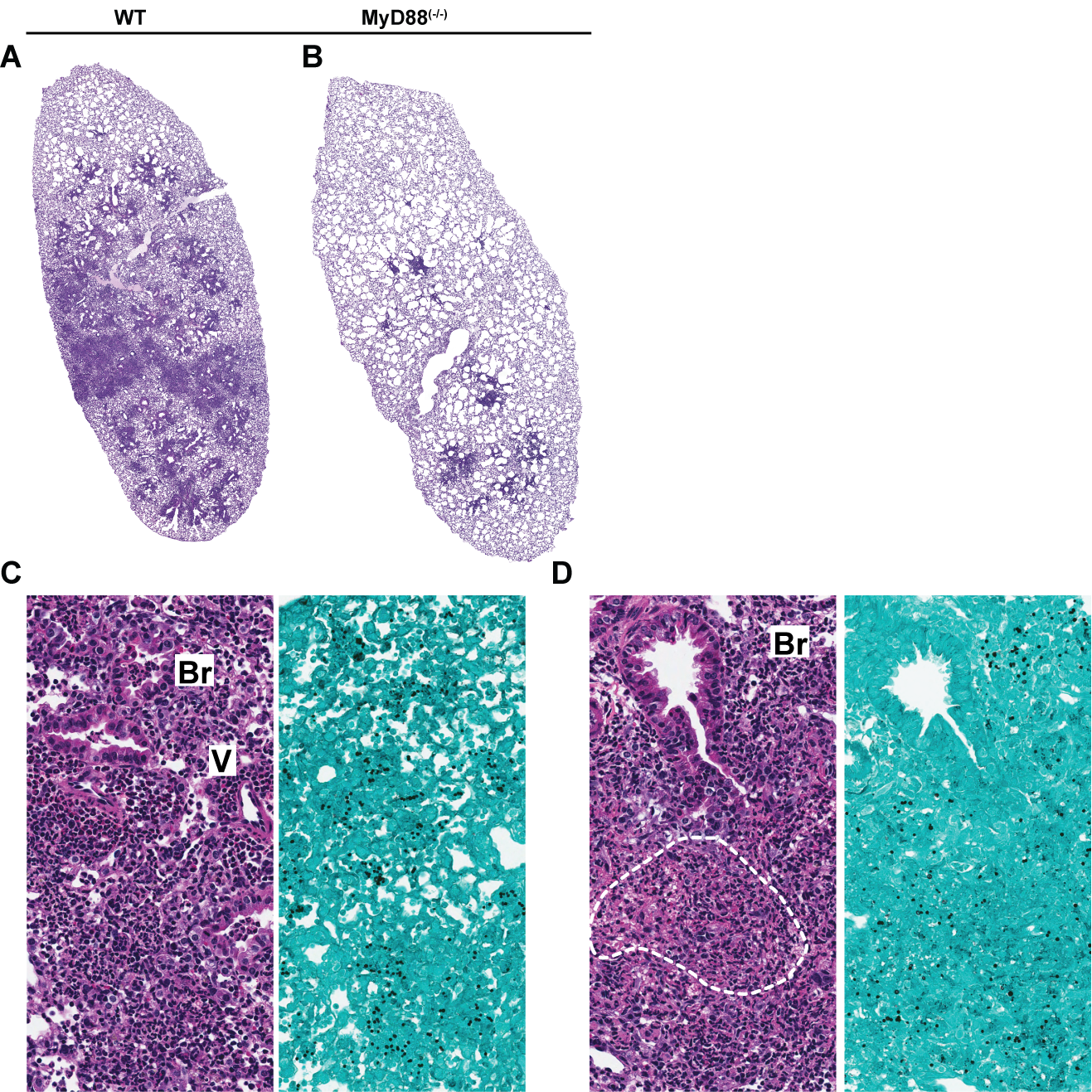
**

**Figure S2 (Supplementary to Figure 2)**

**
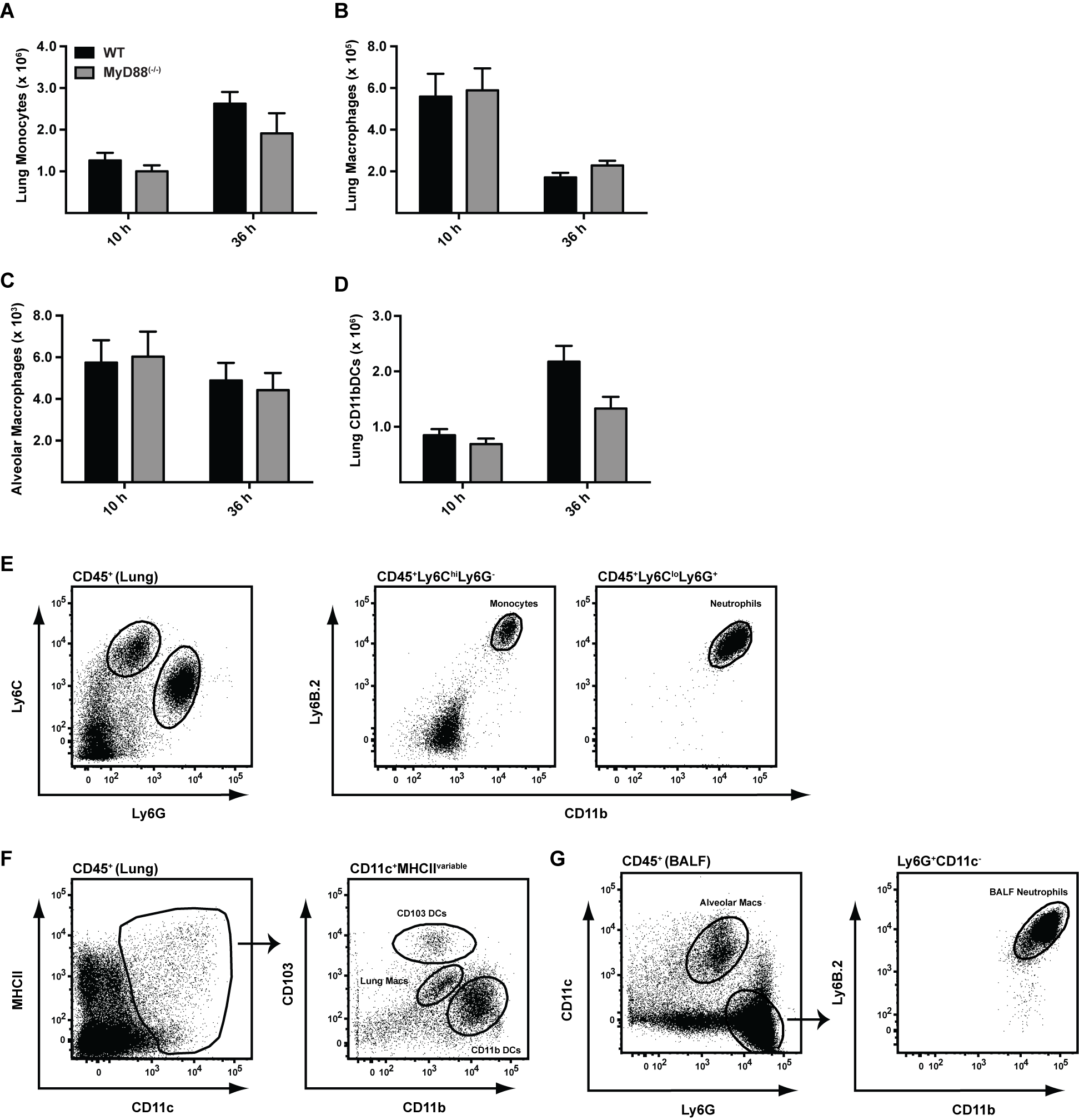
**

**Figure S3 (Supplementary to Figure 2)**

**
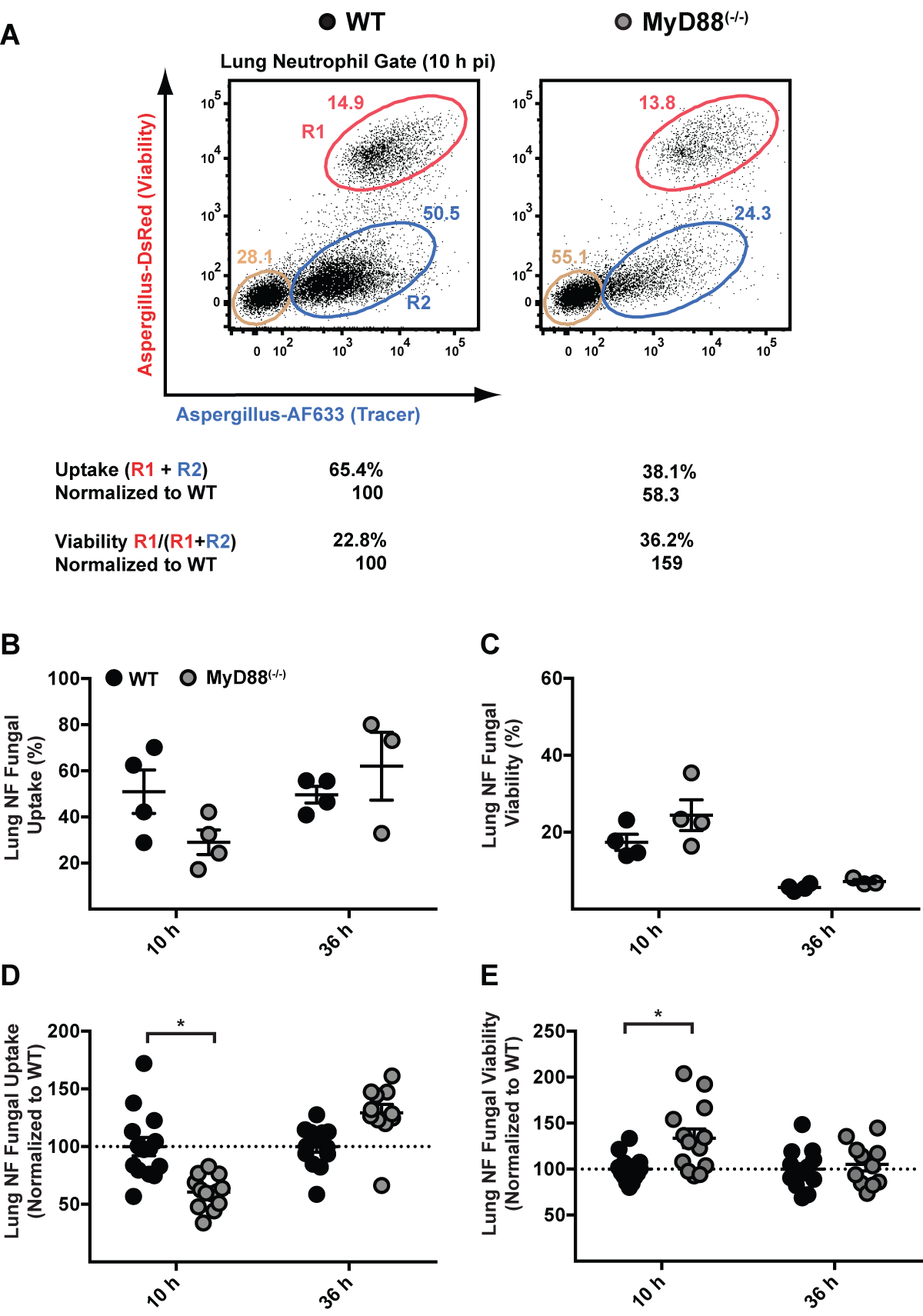
**

**Figure S4 (Supplementary to Figure 2)**

**
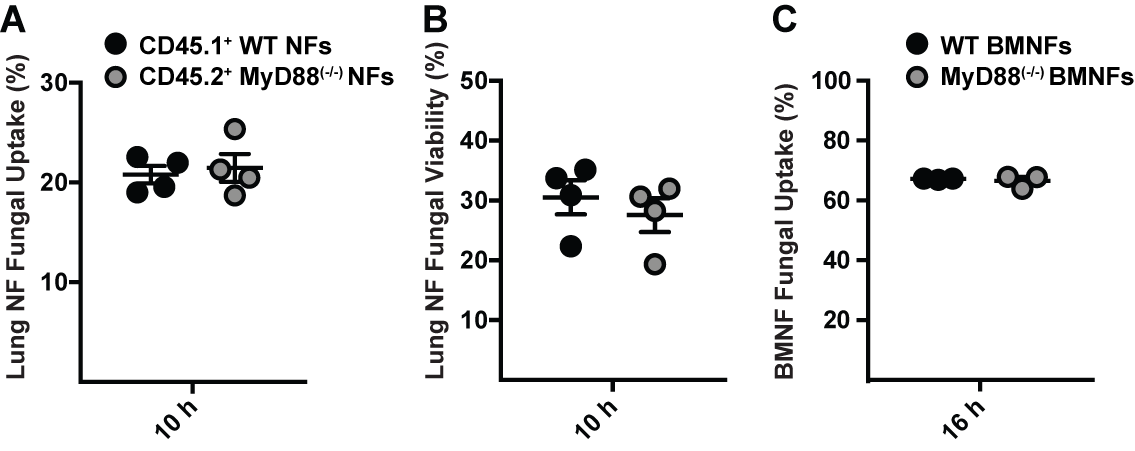
**

**Figure S5 (Supplementary to Figure 3)**

**
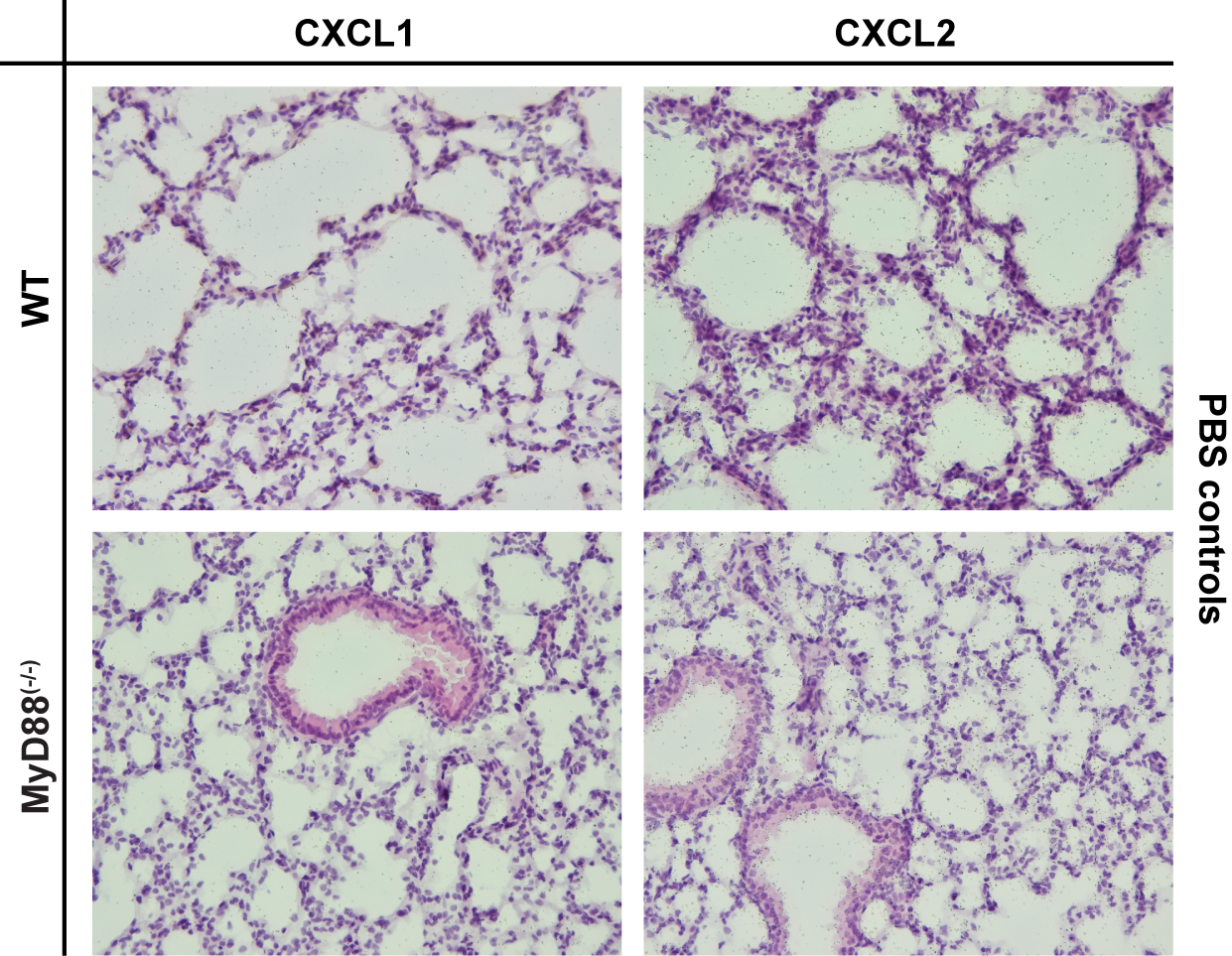
**

**Figure S6 (Supplementary to Figure 3)**

**
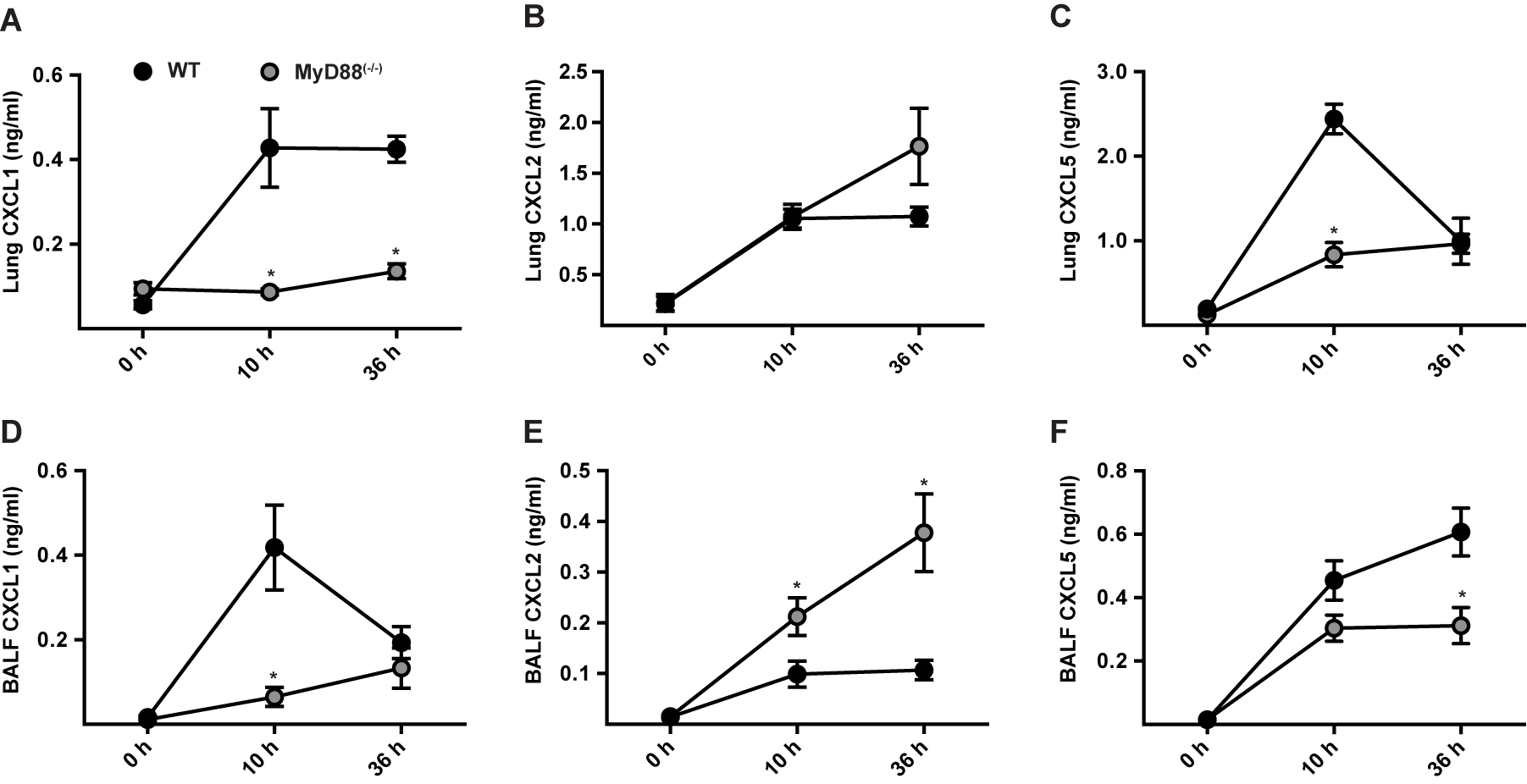
**

**Figure S7 (Supplementary to Figure 4)**

**
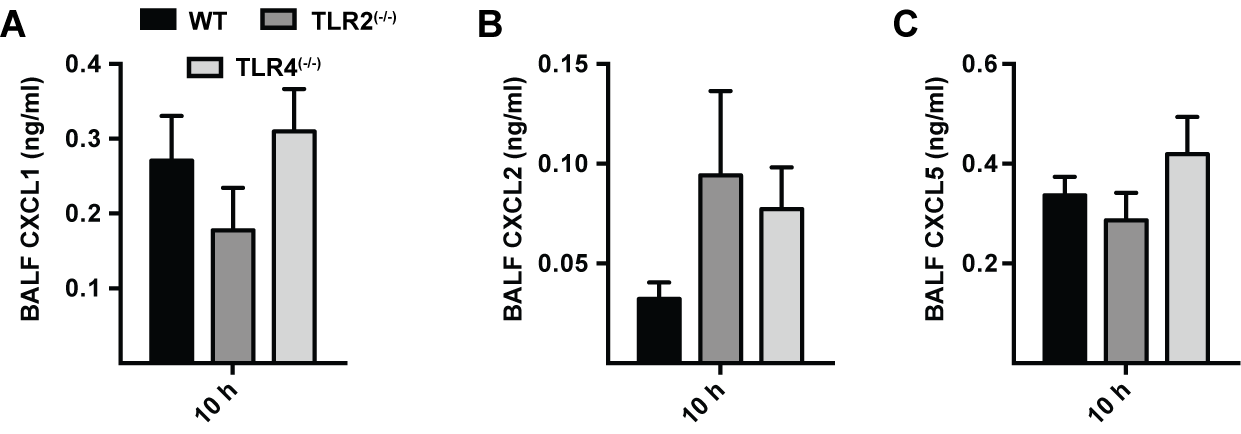
**

**Figure S8 (Supplementary to Figure 4)**

**
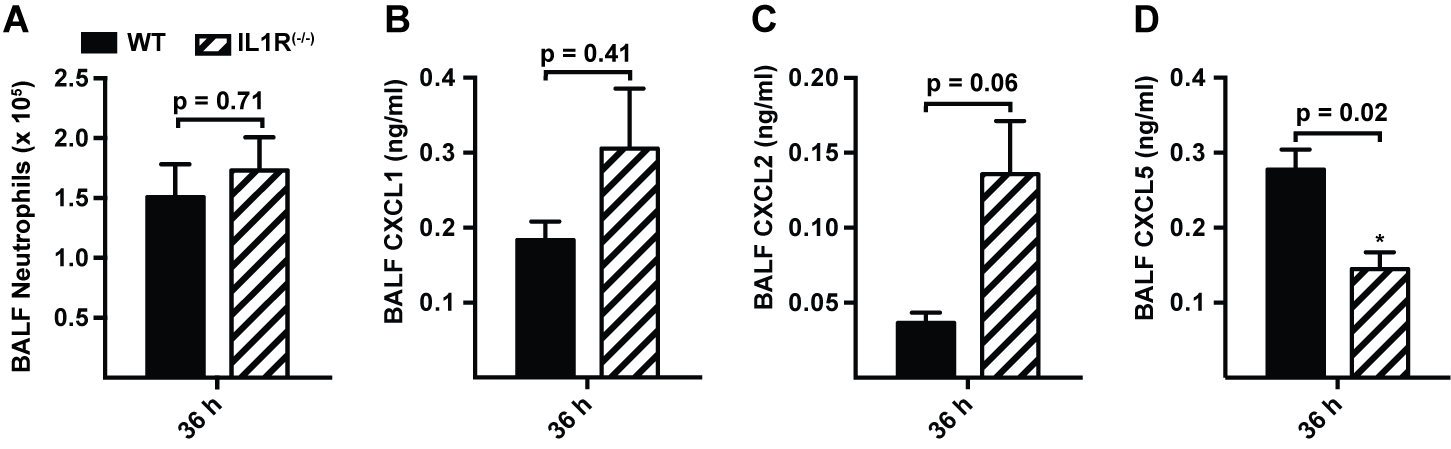
**

**Figure S9 (Supplementary to Figure 5)**

**
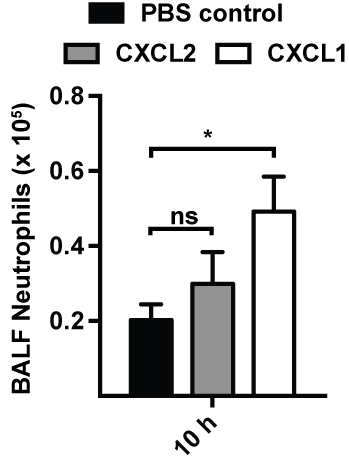
**

**Figure S10 (Supplementary to Figure 5)**

**
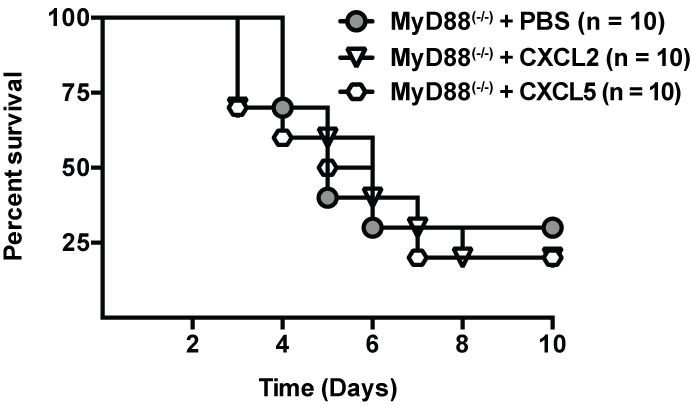
**

**Figure S11 (Supplementary to Figure 8)**

**
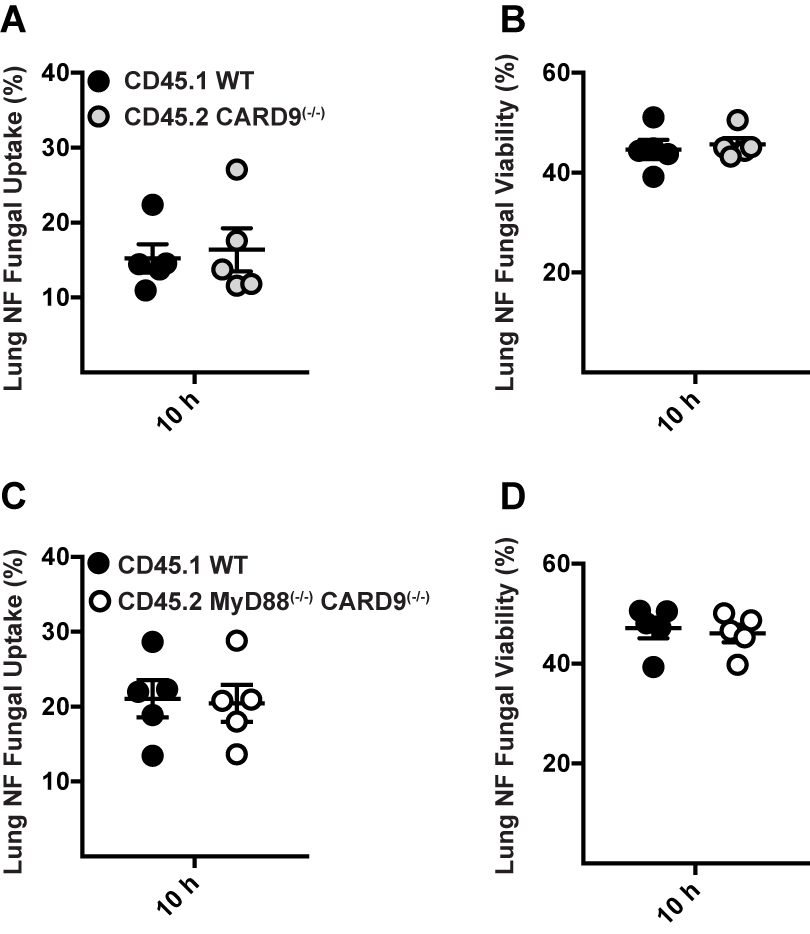
**

**Figure S12 (Supplementary to Figure 8)**

**
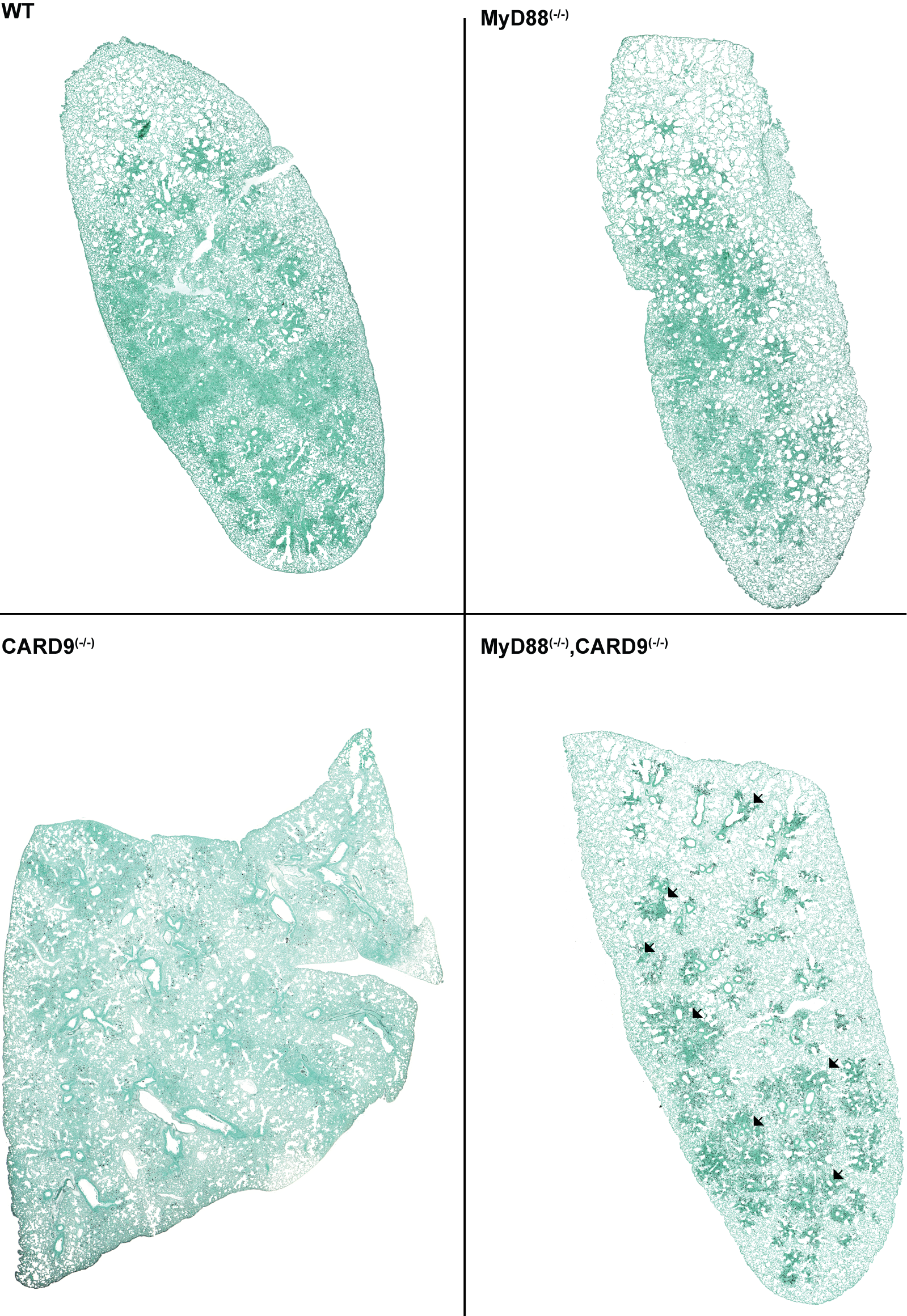
**
