# Supplementary material for: Compartment-Specific and Sequential Role of MyD88 and CARD9 in Chemokine Induction and Innate Defense during Respiratory Fungal Infection
Source: PLoS Pathog. 2015 Jan 26;11(1):e1004589. doi: 10.1371/journal.ppat.1004589 (PMC4306481; doi:10.1371/journal.ppat.1004589)
Supplement: S2 Text — Generation of fluorescent Aspergillus reporter (FLARE) conidia. In vitro Neutrophil Assays. Calculation of Fungal Uptake and Viability in Leukocytes. (DOCX) [file ppat.1004589.s002.docx]

**Text S2**

**Supplemental Materials and Methods**

Generation of fluorescent *Aspergillus* reporter (FLARE) conidia

*A. fumigatus* strain 293 (Af293) was modified to express dsRed as described previously [[1](#_ENREF_1)]. For AF633 labeling, 5 x 10^8^ Af293-dsRed or Af293 conidia (in 1 ml) were biotinylated with 0.5 mg biotin (Biotin XX, SSE; B-6352, Invitrogen), washed and incubated for 30 min at RT with 0.02 mg/ml AF633-streptavidin (S-21375, Invitrogen). Labeled conidia were resuspended in PBS, 0.025% Tween-20 and stored on ice until use.

*In vitro* Neutrophil Assays

For fungal uptake assays, 2 x 10^6^ BM cells were incubated with 2 x 10^6^ FLARE conidia in 2 ml RP10 (RPMI + 10% FBS) at 37^o^C. The frequency of AF633^+^ neutrophils was assessed by flow cytometry a t = 16 h.

Calculation of Fungal Uptake and Viability in Leukocytes

Fungal uptake and fungal viability were calculated as described in [[1](#_ENREF_1)]. In all experiments, fungal uptake refers to the frequency of fungus-engaged neutrophils (dsRed^+^AF633^+^ + dsRed^-^AF633^+^) and fungal viability refers to the frequency of leukocyte that contains live conidia (dsRed^+^AF633^+^) among all fungus-engaged neutrophils.

To facilitate experimental comparisons, the average value for fungal uptake and viability was set to 100 for WT leukocytes and calculated for each sample in an experiment. Data from all experiments were pooled for statistical analysis.

**Supplemental References**

1. Jhingran A, Mar KB, Kumasaka DK, Knoblaugh SE, Ngo LY, et al. (2012) Tracing conidial fate and measuring host cell antifungal activity using a reporter of microbial viability in the lung. Cell Rep 2: 1762-1773.
